# Explicit encoding of temporal dynamics for interpretable melody similarity modeling: insight into machine learning models

**DOI:** 10.3389/fncom.2026.1773922

**Published:** 2026-08-12

**Authors:** Yilin Zhang

**Affiliations:** School of International Arts, Dalian University of Foreign Languages, Dalian, China

**Keywords:** explainable music machine learning, melody similarity, modeling, music information retrieval, recurrent and ensemble models, temporal feature engineering

## Abstract

**Introduction:**

Temporal melody similarity is a fundamental problem in music modelling, and current methods are mostly based on recurrent or attention-based architectures that implicitly learn sequential structures.

**Methods:**

This work adopts an explicit feature-representation approach using temporal-style descriptors, including first-order deltas, relative ratios, polynomial interactions, and rolling statistical features. These features are represented in a model-agnostic manner to enable controlled comparison, interpretability, and reproducible evaluation across architectures. Seven model families were evaluated, including linear baselines, ensemble methods, recurrent networks, attention-based models, and dense fusion architectures, using a large-scale melody similarity dataset containing over 11,000 samples. Cross-validation and bootstrap-based uncertainty estimation were applied for performance evaluation.

**Results:**

Results show that the engineered temporal feature space exhibits significant nonlinearity, with the highest performance achieved by Deep GRU (*R*^2^ = 0.863, MAE = 0.058), followed by XGBoost among non-recurrent models (*R*^2^ = 0.824, MAE = 0.091). Baseline models showed lower performance, demonstrating the nonlinear characteristics of the task. Feature attribution analysis identified temporal descriptors and embedding-based variables as the most influential features.

**Discussion:**

The results demonstrate that TFR provides an interpretable and reproducible foundation for evaluating future sequence-learning architectures, particularly for data-constrained or deployment-oriented applications.

## 1 Introduction

The field of music informatics has emerged quickly at the cross of computational arts, signal processing, and artificial intelligence. Improvements in machine learning and deep learning have enabled the analysis of musical content with a growing degree of accuracy, such as genre classification, music recommendation, symbolic composition, emotion recognition, and similarity retrieval. Among them, melody feature extraction and melody similarity analysis stand out as the core since melodic structure contains valuable information regarding musical identity, progression, and expressive variation. Yet melody is a time thing. The evolution of motifs, the alteration of the contours of the pitch, the development of rhythmic patterns, the emergence of expressive differences are often based on the relative change, and is not associated with the isolated values of features. Thus, to model the similarity of melodies efficiently, there should be the description of the musical characteristics of both melodies at a certain point, and the modeling of the evolution of both musical characteristics over time (Wei et al., 2025; Bontempi et al., 2023). The initial music information retrieval (MIR) systems were based primarily on handcrafted acoustic descriptors, such as Mel-frequency cepstral coefficients (MFCCs), chroma features, pitch-related descriptors and spectral statistics. These features were often used with traditional learning models such as linear classifiers, decision trees, support vector machines, and shallow ensemble methods. These methods provided the ability to be computationally efficient and offer some degree of interpretability, but their performance was often sensitive to the characteristics of the data, the design of features and evaluation procedures. As the deep learning model developed, convolutional neural networks (CNNs), such as long short-term memory (LSTM) and gated recurrent unit (GRU) models, became popular to extract sequential dependencies in the data of music. These models have been shown to be effective on a number of MIR tasks, though they have often needed large datasets, a careful choice of architecture, and extensive hyperparameter optimization, which can limit their usage in data-constrained or deployment-oriented environments (Guter, 2025). Recently still, ensemble learning and attention-based architectures have added to the methodological options that can be used to analyse music and audio. XGBoost and other tree-based models are useful with structured and engineered sets of features because they are capable of modeling nonlinear relationships, heterogeneous feature spaces, and offer feature-importance-based interpretation. Transformer-based models, in contrast, leverage attention mechanisms to capture non-recurrent long-range dependencies and have demonstrated a high potential in both symbolic music generation and sequence modelling. Nevertheless, there are two methodological problems that are not well covered within the existing MIR literature. Temporal modeling is usually viewed as a problem primarily of architecture where recurrent or attention-based models are anticipated to deduce temporal structure based on the input representation. Such traditional temporal descriptors as deltas or delta–delta coefficients are often employed as auxiliary features in this environment and are not a systematic way of describing melodic development. Second, there exist only a limited number of studies that make comparisons between recurrent, ensemble, convolution-inspired, and attention-based models under a shared feature space and unified evaluation protocol. Consequently, it is hard to conclude whether performance differences observed are due to model architecture, feature representation, model capacity, or tuning strategy (Pesek et al., 2017; Benedict et al., 2025).

To solve these problems, this paper considers temporal modeling as a feature-representation problem and not as an architectural learning problem. The proposed temporal-style feature engineering framework explicitly encodes first-order deltas, relative ratios, polynomial interactions, and rolling statistical descriptors to encode melodic dynamics. Contrary to traditional applications of frame-level deltas as auxiliary inputs, the current framework encodes these descriptors as a model-agnostic and structured temporal representation. The design enables the temporal information to be made available to recurrent, ensemble, convolution-inspired and attention-based models in a similar way (Zhang, 2020). Thus, the paper does not assert that temporal feature engineering can be used instead of sequence models, but it investigates the hypothesis whether explicit temporal representation will be able to reduce the reliance on architecture-specific temporal learning and improve interpretability under data-constrained settings. The methodological contribution of this paper is repositioning the temporal descriptors as an important part of the melody similarity modeling.

The framework allows a controlled comparison of various model families on the same spectral, acoustic, and embedding-based inputs, which are by encoding relative change, short-term variation, and mid-term evolution of features into the structured feature space. This is significant in MIR application whereby large annotated datasets, access to raw audio or high-level computation might not always exist. It also helps achieve more transparent analysis as engineered temporal features can be analyzed using feature-importance and attribution methods, but such interpretations should be regarded as complementary and must be tied to musical phenomena with care. The principal findings of this research can be summed up as follows.

- Temporal representation framework: the current study proposes a temporal-style feature engineering framework that is explicitly encoded to encode melodic dynamics using deltas, relative ratios, polynomial interactions and rolling statistical summaries. The framework builds upon the traditional frame-level temporal descriptors, by structuring them into a systematic representation of short- and mid-term melodic variation.
- Model-agnostic comparative design: in the study, Deep GRU, Bi-GRU, XGBoost, a CNN-inspired multi-branch dense network, and a GPT-style Transformer Decoder are evaluated under a single experimental condition. This design facilitates a more tightly regulated comparison of the usage of the same temporally enriched feature space by different model families.
- Interpretable and deployment-oriented analysis: the research questions whether explicit temporal features can make the similarity model of melody modeling accurate and interpretable under data-constrained conditions. In order to investigate influential descriptors, the analyses based on feature-importance and SHAP are carried out, and the results are interpreted carefully in the context of model architecture, dataset size, and task characteristics.
- Applicability of MIR systems practical relevance: the proposed framework will be used in music similarity, recommendation, and retrieval context where reproducibility, interpretability, and computational efficiency are key attributes. The approach offers a viable foundation on which to compare lightweight and deep learning models in structured music analytics by shifting part of the temporal modeling burden off of architecture design on to feature representation. On the whole, the study strives to offer a reproducible and interpretable model to analyse melody similarity by integrating explicit representation of temporal features with controlled cross-model testing.

The results are not supposed to provide universal rankings of model families, but simply clarify the interaction of different architectures with temporally engineered features of the current experimental environment. This conservative framing is significant to support reasonable methodological acumen and to guide future MIR research with larger datasets, alternative similarity-label construction techniques, and more extensive hyperparameter controls.

Compared to existing MIR approaches that rely primarily on architectural design such as recurrent or attention-based networks to infer temporal structure implicitly from raw or minimally processed inputs, the proposed framework differs in that temporal dynamics are explicitly encoded at the feature level through a structured and model-agnostic representation layer. This design choice allows the same temporally enriched feature space to be shared across recurrent, ensemble, dense-fusion, and attention-based model families, enabling a more controlled and interpretable comparison than is typically possible when each model family operates on a different input representation. Furthermore, unlike most existing studies that report performance for a single model family or architecture, this work evaluates five structurally distinct model families under identical experimental conditions, providing a broader empirical basis for assessing the contribution of temporal feature engineering relative to architectural capacity. Nevertheless, several limitations of the proposed framework must be acknowledged alongside these contributions. The similarity target was constructed using embedding-based cosine similarity rather than human perceptual judgements, which limits the extent to which findings can be interpreted as reflective of listener-perceived melodic similarity. The dataset was assembled from processed descriptors derived from publicly available sources, and results may therefore not generalise fully across genres, instruments, or recording conditions. In the study, fold-wise cross-validation results including 95% confidence intervals are reported for all seven compared model families, providing a complete statistical basis for model comparison.

## 2 Related work and literature review

The more recent research in the field of melody analysis has compared the various methods of modeling that encompassed hand-crafted pipelines of features, deep end-to-end architectures, as well as multimodal fusion frameworks. The hybrid CNN-RNN models have been extremely successful at classification tasks based on spectrograms, whereas models based on transformers have been able to capture long-term musical structure very well, especially in situations of symbolic representation (Senatori et al., 2025). Meanwhile, ensemble methods still stand their own when applied on the features that have been carefully designed, especially in regression and similarity-based tasks. However, most of the existing literature only reports single paradigms of features design and various learning architectures, making it difficult to analyze the relationship between feature design and various learning architectures. Furthermore, there are seldom explicit comparisons of temporal feature engineering to temporal modeling in architecture. The present study provides the scientific community with the knowledge that temporal-style features can be used as a common representation over diverse model families as shown in Table 1.

**Table 1:** Recent research in melody feature extraction and similarity analysis.

Study/source	Methodology	Key contribution	Reported performance
Wang et al. (2024)	Survey of MIR evaluation methods	Identified reproducibility and evaluation issues in shallow feature approaches	Highlighted limitations in genre classification
Son (2021)	RNN Encoder–Decoder	Introduced recurrent modeling for sequential data	Improved sequence representation
Alqudah and Moussavi (2025)	CNN + RNN hybrid	Applied deep learning to spectrogram-based genre classification	Accuracy > 90% (GTZAN)
Fuhg et al. (2025)	XGBoost ensemble	Robust modeling of engineered features with regularization	Competitive regression performance
Wang and Bao (2020)	Transformer attention	Long-range dependency modeling without recurrence	State-of-the-art in NLP, adapted to music
Yang et al. (2025)	Music Transformer	Long-term symbolic music generation	Coherent multi-bar musical structure
Pacha et al. (2018)	Multimodal fusion (lyrics + audio)	Improved genre recognition via multimodal learning	Demonstrated gains over unimodal models

**Figure 1:**
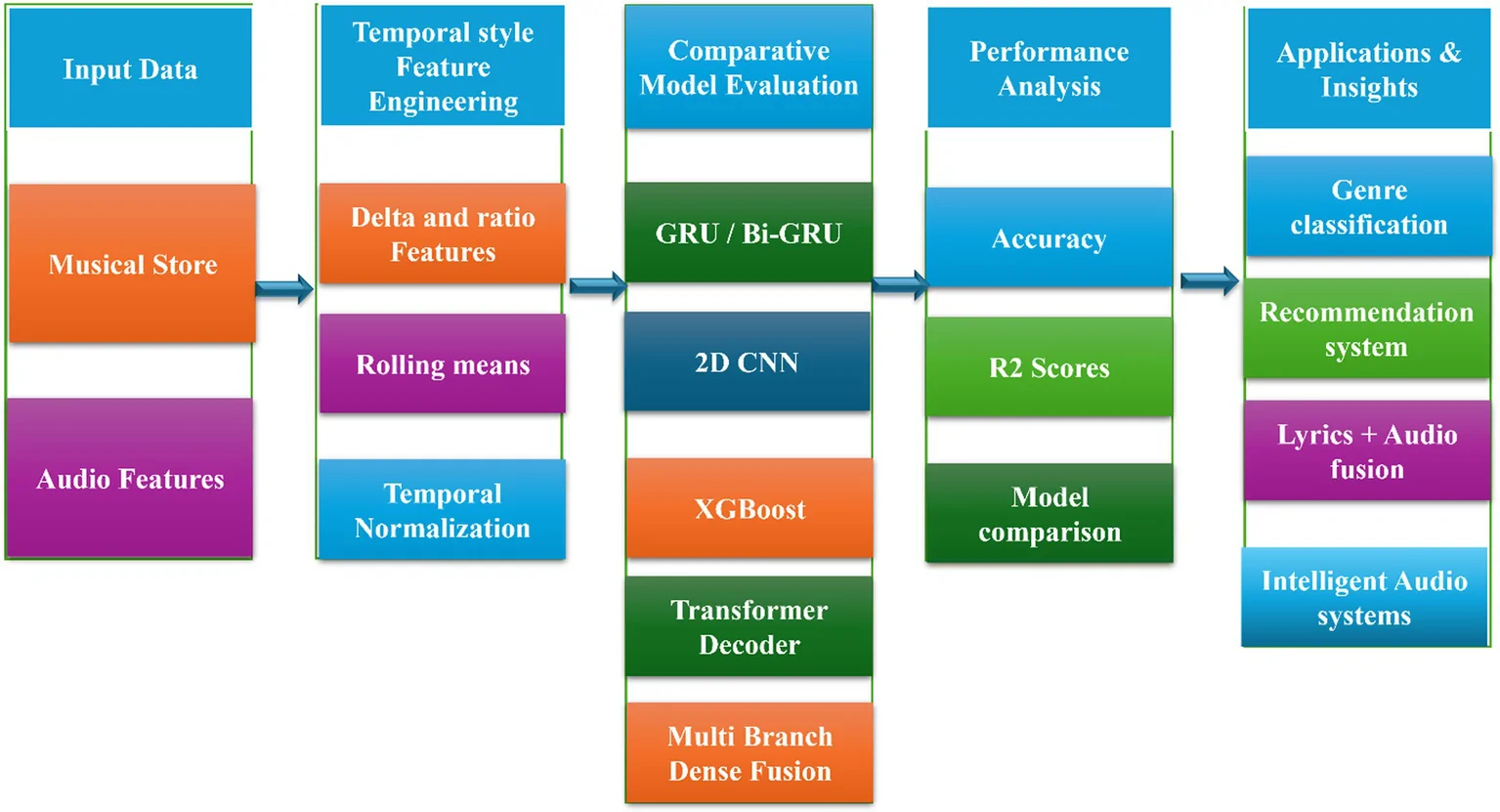
Schematic illustration of the proposed temporal-style feature engineering framework and comparative model evaluation pipeline for melody similarity analysis.

To have a more comprehensive overview and the entire structure, Figure 1 shows the overall structure of the proposed research. The figure depicts the process of gradual passing of input of musical data and construction of temporal-style features to modeling comparison of recurrent, ensemble, and transformer-based architectures and then performance and analysis based on applications.

The originality of this work is that it repositions temporal feature engineering as a first-class modeling component, as opposed to a preprocessing auxiliary step. Rather than merely depending on architectural mechanisms like recurrence or attention to deduce temporal structure, this paper explicitly encodes the development of spectrometric and learned embedding features through temporal-style transformations. In this way, one can methodically research the various ways in which model families can benefit by the same time-based representations, thus bridging a gap in the current MIR literature in which feature design and model inductive bias are rarely considered together (Park et al., 2025; Leprince et al., 2023).

## 3 Methodology

The methodology was constructed in such a way that it compared whether explicitly encoded temporal-style descriptors can enhance the modeling of melody similarity in data-constrained and reproducibility-oriented conditions. The paper had seven primary steps: dataset preparation, feature representation, similarity-target construction, temporal-style feature engineering, baseline and ablation design, comparative model evaluation, and interpretability. The proposed framework, as compared to end-to-end pipelines of raw-audio, had been pre-extracted and harmonized numerical music descriptors. This design enabled recurrent, ensemble, attention-based, and dense-fusion models to be evaluated with the same structured input space, eliminating the chance that differences in performance were due to differences in feature availability.

### 3.1 Dataset description

The experimental dataset was built on publicly available music information retrieval datasets and benchmark sources, including GTZAN, FMA, MIR-1K, and the million song dataset (Table 2). These sources were chosen due to their provision of commonly-used audio, metadata, symbolic, and embedding-related information in MIR research. The datasets were not utilized on their entire original scale. Instead, they were used as source corpora where compatible feature representations were selected, cleaned, harmonized and reorganized into a single tabular structure to model melody similarities. The end experimental dataset consisted of 11,000 processed instances. Every case comprised of fixed acoustic descriptors and time extended descriptors. The final feature space had about 76 engineered features variables following temporal-style expansion. The data were presented in a tabular format, and thus the raw audio files were not needed when training the model. It was selected to enhance reproducibility and to enable a controlled comparison between different model families.

**Table 2:** Public music datasets used as source references for feature construction and dataset assembly.

Source	Dataset name	Data type	Approximate original size	Features used or adapted	Reference/DOI
Public dataset	GTZAN	Music genre audio clips	1,000 clips	MFCCs, spectrogram descriptors, pitch-related features	10.1109/TSA.2002.800560
Public dataset	FMA (free music archive)	Audio tracks and metadata	106,574 tracks	MFCCs, spectral descriptors, metadata, embedding-based features	10.48550/arXiv.1612.01840
Public dataset	MIR-1 K	Singing voice and music clips	1,000 clips	Pitch contour, MFCCs, vocal/accompaniment-related descriptors	10.1109/TASL.2009.2026503
Public dataset	Million song dataset	Audio features and metadata	More than 1,000,000 tracks	Metadata and high-level audio/embedding descriptors	10.1109/ICASSP.2011.5947637

The sample sizes in Table 2 are the full public data sets, but only the harmonised processed subset was used in the modeling experiments. To enhance the level of transparency, the final dataset preparation only included records whose descriptors were in compatible formats and excluded incomplete, duplicated and near-duplicated records.

### 3.2 Feature representation

Low-level, mid-level and high-level descriptors were used to represent each sample. The low-level acoustic features were spectrogram descriptors and Mel-frequency cepstral coefficients (MFCCs), which reflected a time-frequency energy allocation and timbral properties. Melodic movement and fundamental-frequency variation as indicated in Table 3 were described using pitch-contour characters (Safdar et al., 2024). MIDI-index features were used wherever possible to represent symbolic information. Deep embedding functions were added as small latent representations of the higher-level musical structure.

**Table 3:** Main feature groups used in the proposed framework.

Feature group	Description	Role in melody similarity modeling
Spectrogram descriptors	Time–frequency representation of audio energy	Capture acoustic texture, local spectral structure, and energy distribution
MFCC features	Mel-frequency cepstral coefficients	Represent timbral and low-level acoustic characteristics
Pitch contour	Fundamental-frequency trajectory	Capture melodic movement, interval direction, and tonal progression
MIDI index	Symbolic note/event-related information	Provide structured symbolic cues where available
Deep embeddings	Latent representations from pretrained or learned models	Capture higher-level musical and acoustic relationships
Temporal-style descriptors	Deltas, ratios, polynomial interactions, and rolling statistics	Encode short-term variation, proportional change, and mid-term melodic development

All continuous features were normalized, and the models were trained. To put heterogeneous features in a similar numerical range, Min-Max scaling was applied to them. Descriptors related to MFCC were also standardized where necessary due to the possibility of varying distributions of these types of descriptors compared to symbolic or embedding-based variables. Before temporal expansion, missing values, duplicate records and inconsistent entries of features were eliminated.

### 3.3 Target variable construction

Melody similarity was formulated as a continuous regression problem. The target variable represented the degree of similarity between two musical samples and was computed using cosine similarity between their embedding vectors. For two samples represented by embedding vectors *Ei* and *Ej*, the similarity score was calculated as:

Similarity(Ei,Ej)=∥Ei∥∥Ej∥Ei·Ej

Where *Ei* and *Ej* are the embedding vectors of two musical samples, *Ei Ej* is their dot product, and ∥*Ei*∥ and ∥*Ej*∥ are their vector norms. This formulation allowed melody similarity to be treated as a continuous prediction target rather than as a discrete classification label.

To reduce information leakage, target-derived variables were not used as direct predictors unless they were part of the predefined embedding representation before similarity-score construction. Overlapping or near-duplicate samples were also removed during preprocessing.

### 3.4 Temporal-style feature engineering

The main methodological approach was the creation of temporal-style descriptors. In traditional MIR pipelines, deltas, delta–delta coefficients are typically employed as auxiliary frame-level descriptors as illustrated in Table 4. The following descriptors were introduced into a structured representation layer in this study which also incorporated relative ratios, polynomial interactions and rolling statistics (Liang, 2023). Thus, the proposed temporal-style feature set is not to be interpreted as a completely new signal-processing operation, but rather as a systematic approach to the representation that makes temporal variation available in a variety of model families within the same feature space. The descriptors of the temporal-style were the first-order deltas, second-order deltas, relative ratios, terms of polynomial interaction, rolling means and rolling variances. These changes were meant to reflect short-term change, local acceleration, proportional variation, nonlinear interaction and mid-term melodic stability.

**Table 4:** Temporal-style feature groups and their methodological purpose.

Temporal feature group	Example operation	Main purpose	Musicological interpretation
First-order deltas	Δ_xt_ = x_t_ − x_t−1_	Capture short-term change	Local pitch movement, spectral change, melodic direction
Second-order deltas	Δ^2^x_t_ = Δx_t_ − Δx_t−1_	Capture acceleration or curvature in feature evolution	Change in melodic motion or expressive transition
Relative ratios	rt = xt/(xt-1 + *ε*)	Encode proportional variation	Relative energy, pitch, or spectral fluctuation
Polynomial interactions	$p_{t}=x_{t}\cdot x_{\left\{t-1\right\}}\cdot 2x_{t}^{2}$	Capture nonlinear feature relationships	Interaction between pitch, timbre, and embedding descriptors
Rolling mean	*μ*_t_ over local windows	Represent local stability	Sustained melodic or timbral patterns
Rolling variance	*σ*_t_^2^ over local windows	Represent local variability	Phrase-level variation or expressive fluctuation

Rolling statistics were calculated with the window length of 5, 10 and 20 frames and the framework was able to capture both the short term and the mid-term melodic behavior. To prevent division by zero a small constant *ϵ* was added to denominations in ratio-based features. Once the time expansion had been done, the original base descriptors were then converted to an approximation of 76 engineered featured space per sample.

### 3.5 Data splitting and leakage prevention

The data set was segregated into training, validation, and testing sets. The primary hold-out analysis employed an 80:20 train-test-split, and a validation subset was selected as part of the training data to rank the models and stop them early. Also, the stability of model performance with respect to data partitions was evaluated by cross-validation 15 times. All the models were subjected to the same preprocessing, scaling, feature-generation procedure and evaluation metrics. The prevention of leakages was dealt with on three levels. First, variables derived by targeting were not included in the input features (Schedl, 2019). Second, before splitting, duplicate and near-duplicate samples were eliminated. Third, in cases where metadata were available, samples based on the same source track were stored in the same partition to minimize the possibility of segment-level leakage. This was done in order to minimize the chances that performance on the test-sets was due to memorization of repeated or overlapping musical material and not generalization to unseen samples. Since not all publicly available datasets provide complete artist- or segment-level metadata in a harmonized format, the split was largely controlled on the sample and source-track level on which such metadata were present. This has been recognized as a limitation and future work should further prove the framework using fully song independent, artist independent and corpus independent splits.

### 3.6 Baseline and ablation design

To directly evaluate the contribution of temporal-style feature engineering, two main feature configurations were defined. The first setup only used static features, such as spectrogram descriptors, MFCCs, pitch-contour features, MIDI-index features, and deep embeddings. The second design incorporated the same static characteristics and the suggested temporal-style characteristics. This design enabled the study to approximate the net gain of explicit temporal representation of each model family (Table 5).

**Table 5:** Feature configurations used for baseline and ablation analysis.

Configuration	Included features	Purpose
Static-feature baseline	Original spectral, MFCC, pitch, MIDI, and embedding descriptors	Establish model performance without temporal-style expansion
Temporal-style feature set	Static features plus deltas, ratios, polynomial interactions, rolling means, and rolling variances	Evaluate the added value of explicit temporal representation
Ablation variants	Temporal-style feature set with selected groups removed	Examine the contribution of deltas, ratios, rolling statistics, and interaction terms

This design explicitly decomposes the contribution of feature representation and that of model architecture. Besides the baseline of static- feature, ablation experiments were also conducted to understand what impact the removal of the key temporal-feature groups.

### 3.7 Model design and comparative evaluation

Five model families were tested through the same experimental framework: Deep GRU, Bi-GRU, XGBoost, a GPT-style Transformer Decoder, and a CNN-inspired multi-branch dense fusion network. These models have been chosen because they reflect the various inductive biases such as recurrent temporal memory, bidirectional temporal context, structured ensemble learning, attention-based global dependency modeling, and localized feature-group fusion (Jones and Friberg, 2023).

#### 3.7.1 Deep GRU

A stacked gated recurrent unit model was used as the main recurrent architecture. The network had three GRU layers having hidden sizes of 256–256-128, and a dense regression head. To decrease overfitting and stabilize training, dropout and batch normalization were used. GRU model was added since gated recurrent architectures have the ability to learn sequential dependencies between temporally grouped feature inputs. A detailed implementation-oriented summary of the Deep GRU workflow, including feature engineering, normalization, sequence shaping, optimizer selection, callbacks, and training steps, is provided in Appendix Table A1.

#### 3.7.2 Bi-GRU

A bidirectional GRU model was added to test whether forward and backward temporal context enhance prediction in the case of implicitly encoded predictive information in the feature space. The Bi-GRU incorporates contextual dependencies that might not be adequately reflected by a unidirectional recurrent model.

#### 3.7.3 XGBoost

XGBoost has been adopted as non-recurrent structured learning model. It builds a collection of regression trees via gradient boosting and is able to identify nonlinear associations between created features. To reduce overfitting, regularization, shrinkage, subsampling, and control of tree-depths were employed. Since XGBoost does not internally enforce a temporal ordering on the internally represented data, its performance indicates the extent of the already present temporal information already captured by the engineered feature space. The main XGBoost techniques used in this study, including gradient boosting, regularization, shrinkage, subsampling, tree-depth control, missing-value handling, and early stopping, are summarized in Appendix Table A2.

#### 3.7.4 GPT-style transformer decoder

To test attention-based modeling of temporally enriched structured inputs, a Transformer Decoder was implemented. The feature vectors were considered sequential tokens and enhanced with positional encoding. Multi-head self-attention was used to capture global dependencies among feature tokens. Transformer model was not a presumed better architecture, but a comparative attention-based baseline (Tien et al., 2022). The implementation components of the GPT-style Transformer Decoder, including feature embeddings, positional encoding, multi-head attention, optimizer strategy, learning-rate scheduling, loss function, and diagnostic evaluation, are summarized in Appendix Table A3.

#### 3.7.5 CNN-inspired multi-branch dense fusion network

An inspiration non-recurrent neural baseline was a multi-branch dense fusion network inspired by CNN-style feature separation. The model had distinct branches of spectrogram related features, deep embedding features and engineered temporal features. Branches processed their feature group on its own before being fused to a common dense regression head. It is not a real 2D convolutional network, but offers a convenient baseline to judge localized feature interactions and domain specific feature fusion. Table 6 summarizes the model-comparison design.

**Table 6:** Summary of model families and comparative role.

Model	Learning paradigm	Main input form	Comparative role
Deep GRU	Recurrent neural model	Temporal feature windows	Tests sequential memory with explicit temporal features
Bi-GRU	Bidirectional recurrent model	Temporal feature windows	Tests the added value of forward–backward temporal context
XGBoost	Tree-based ensemble model	Structured tabular features	Tests whether engineered temporal features are informative without recurrence
Transformer decoder	Attention-based model	Tokenized temporal feature vectors	Tests global attention-based dependency modeling
Multi-branch dense network	CNN-inspired dense fusion model	Feature-group branches	Tests localized feature interaction and feature-group fusion

### 3.8 Training strategy and evaluation protocol

All models were trained as supervised regression models to predict melody similarity. To ensure consistency of the different model architectures, the main optimization goal was the Mean Squared Error (MSE). To prevent overfitting and stability issues in convergence, early stopping and learning-rate scheduling strategies were used for training neural network models. Regularization, shrinkage, subsampling, tree-depth control and stopping based on validation were used to enhance generalization performance in the XGBoost model. The performance of models was assessed using four widely-used regression metrics: coefficient of determination (R^2^), Mean Absolute Error (MAE), Mean Squared Error (MSE), and Mean Absolute Percentage Error (MAPE). These measures reflect various aspects of predictive accuracy such as explained variance, average prediction error, size of squared error and relative prediction error. The following evaluation measures were used

$$\mathrm{MSE}=\frac{1}{n}\Sigma_{i=1}^{n}{\left(y_{i}-{\hat{y}}_{i}\right)}^{2}$$

$$\mathrm{MAE}=\frac{1}{n}\Sigma_{i=1}^{n}|y_{i}-{\hat{y}}_{i}|$$

$$R^{2}=1-\frac{\sum_{i=1}^{n}{\left(y_{i}-{\hat{y}}_{i}\right)}^{2}}{\sum_{i=1}^{n}{\left(y_{i}-\overset{-}{y}\right)}^{2}}$$

$$\text{MAPE}=\frac{1}{n}\sum_{i=1}^{n}|\frac{y_{i}-{\hat{y}}_{i}}{y_{i}}|\times 100\%$$

Where *y*ᵢ is the observed similarity value, *ŷ*ᵢ is the predicted similarity value, *ȳ* is the mean observed value, and *n* is the number of test samples.

All models were evaluated using identical preprocessing procedures, feature representations, and evaluation metrics. Model stability under different data partitions was assessed using 15-fold cross-validation. Independent bootstrap resampling was also performed for each model to provide estimates of uncertainty in performance estimation. The performance values were evaluated in each bootstrap replicate, and the final performance values were presented as mean values with 95% confidence intervals based on the percentile bootstrap method.

The percentile bootstrap confidence interval was derived from the empirical distribution function of the values of the metric, instead of the normally distributed and independent fold level estimates. This way, one can overcome the limitation of direct cross validation folds analytical confidence intervals and get a better estimate of the variability due to variations in data sampling and model evaluation. True versus predicted plots, residual box plots, cumulative error plots, and training-validation loss curves were used for the diagnostic performance analyses. The analyses were used to look at agreement in prediction, error distribution, convergence behaviour, and possible overfitting in building the models. Table 7 summarizes the whole training and evaluation process.

**Table 7:** Summary of training and evaluation protocol.

Component	Implementation in this study
Prediction task	Continuous regression of melody similarity
Train–test split	80:20
Cross-validation	15-fold cross-validation
Main optimization objective	Mean squared error
Main evaluation metrics	(*R*^2^), MAE, MSE, MAPE
Neural-model control	Early stopping and learning-rate scheduling
XGBoost control	Regularization, shrinkage, subsampling, tree-depth control
Diagnostic plots	Actual-versus-predicted plots, box plots, cumulative error plots, loss curves
Reporting strategy	Testing-set performance with bootstrap-based 95% confidence intervals

### 3.9 Interpretability and musicological interpretation

To understand the features that had the most significant influence on the prediction of melody similarity, interpretability analysis was performed. In the case of XGBoost, SHAP analysis was applied to determine the importance of features on a global scale and to estimate the effects of a feature. In neural models, permutation feature importance was estimated to estimate the performance loss when single groups of features were disrupted. Since engineered temporal features are correlated, the results of interpretation were considered indicative as opposed to causal. To give a musical basis, deltas of the pitch were taken to mean the characteristics of melodic contour, interval movement, and phrase direction (Ding et al., 2023).

**Figure 2:**
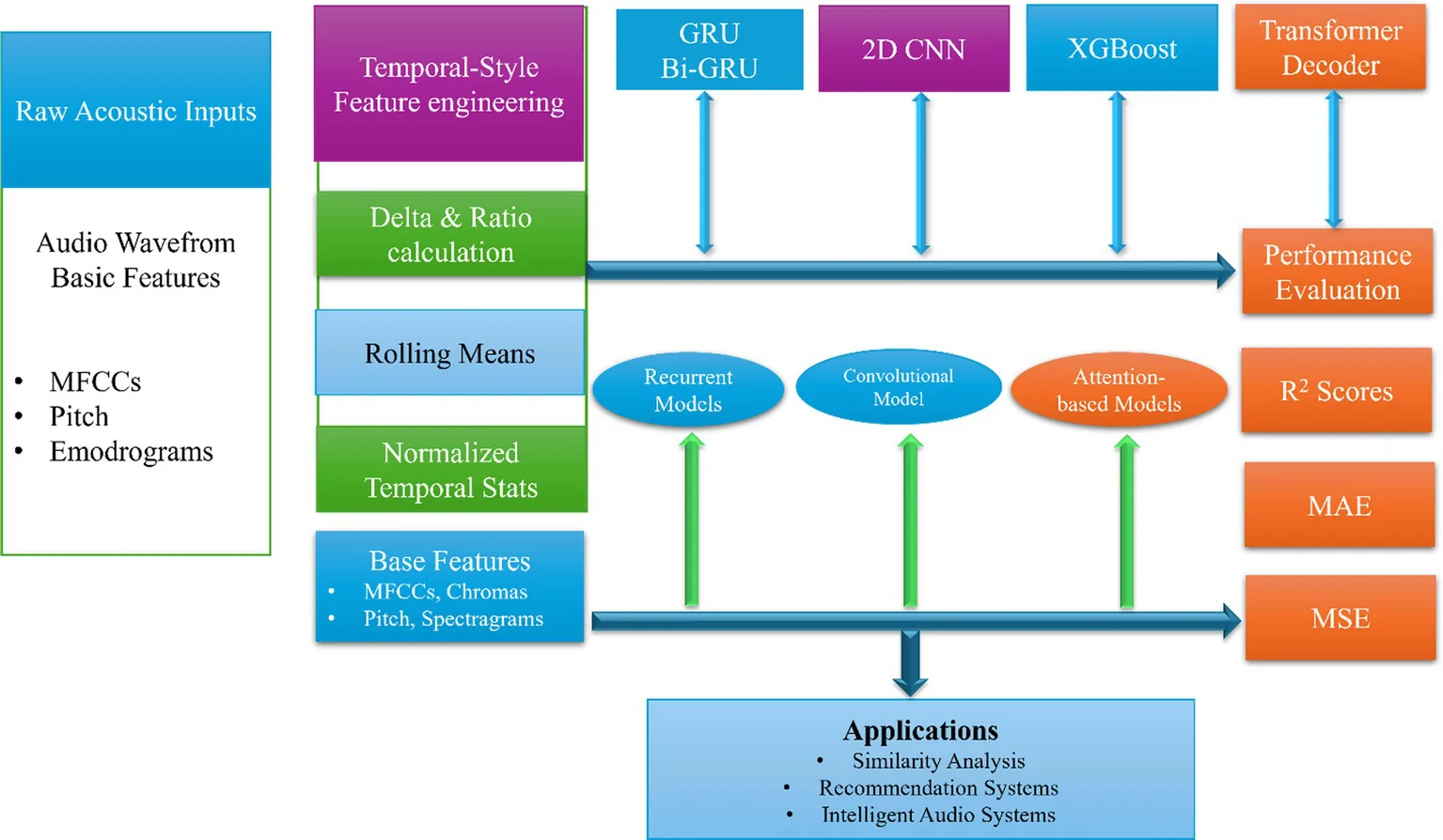
Overview of the proposed temporal-style feature engineering and comparative evaluation framework across multiple model families.

Ratios based on spectrogram and energy were construed as measures of timbral and dynamic fluctuation. Rolling implied and rolling variances were viewed as local stability and recurring patterns and phrase-based variance. Embedding-based deltas were viewed with trepidation as high-level cues to variations in learned musical representation. Therefore, the model inspection and explanation were supported by SHAP and permutation analyses, which did not prove direct causal relationships between individual features and human perception of melodic similarity. The proposed temporal-style feature engineering and comparative evaluation framework across multiple model families is depicted in Figure 2.

### 3.10 Computational environment and reproducibility

All the experiments were carried out in a controlled local computing environment. The experimental platform was a Dell Alienware workstation with an Intel core i7-12700H CPU, a 16GB RAM, an NVIDIA GeForce RTX3060 graphics card with 6GB VRAM, and 512GB of solid-state storage. Structured feature representations were used in the framework instead of computationally expensive raw-audio pipelines that minimized hardware needs and enhanced practical reproducibility. To support reproducibility, the preprocessing procedure, feature-construction steps, model settings, random seeds, train–test split strategy, and processed feature availability should be reported in the final manuscript or Supplementary material. Where feasible, processed features and code must be accessible by a public repository. Unless published publicly, the processed feature matrix, model settings, and scripts are to be made available by the respective author upon reasonable request.

## 4 Results and discussion

In this section, the authors report findings of the compared models in terms of predictive performance, diagnostic behavior, cross-validation stability, and interpretability. These results come in a condensed form in order to facilitate a more justifiable comparison across model families. The results are viewed with great care and are not supposed to provide a universal ranking of recurrent, ensemble, dense-fusion, or attention-based methods.

### 4.1 Overall predictive performance of the compared models

The predictive ability of all the models evaluated is summarized in Table 8, which shows the algorithm evaluated on the extended dataset of 11,000 samples and 76 engineered features. The same preprocessing, feature representation and validation process were used for all models. The uncertainty of the model was estimated through independent bootstrap resampling, and reported values are mean values with the 95% percentile bootstrap confidence interval.

**Table 8:** Performance of all compared models on the extended dataset (11,000 samples, 76 engineered features) evaluated using independent bootstrap-resampled datasets.

Model	*R*^2^ (mean ± SD)	95% CI (*R*^2^)	MAE (mean ± SD)	95% CI MAE	MSE (mean ± SD)	95% CI MSE	MAPE (%) (mean ± SD)	95% CI MAPE (%)	Interpretation
Linear regression	0.2226 ± 0.0483	[0.2047–0.2412]	0.2291 ± 0.0022	[0.2246–0.2324]	0.0818 ± 0.0013	[0.0791–0.0841]	168.12 ± 19.85	[159.12–177.57]	Weak linear baseline; confirms nonlinearity of task
Random forest	0.5893 ± 0.0232	[0.5412–0.6337]	0.1581 ± 0.0049	[0.1488–0.1678]	0.0432 ± 0.0026	[0.0383–0.0482]	125.87 ± 5.95	[116.69–139.60]	Stronger baseline; still substantially below deep models
XGBoost	0.8240 ± 0.0071	[0.8105–0.8376]	0.0913 ± 0.0020	[0.0873–0.0950]	0.0185 ± 0.0008	[0.0171–0.0201]	79.70 ± 4.44	[71.89–89.04]	Best non-recurrent model; strong structured-feature learner
Deep GRU	0.8632 ± 0.0172	[0.8369–0.8855]	0.0576 ± 0.0076	[0.0501–0.0742]	0.0144 ± 0.0018	[0.0120–0.0173]	61.40 ± 10.08	[50.08–83.47]	Best recurrent model; most stable CV across folds
Transformer decoder	0.8240 ± 0.0135	[0.8063–0.8432]	0.0789 ± 0.0038	[0.0733–0.0826]	0.0185 ± 0.0019	[0.0167–0.0203]	84.61 ± 8.99	[76.01–93.80]	Competitive attention model; sensitive to dataset scale
MBDFN	0.6601 ± 0.0355	[0.6348–0.7060]	0.1386 ± 0.0087	[0.1279–0.1456]	0.0357 ± 0.0038	[0.0306–0.0385]	137.43 ± 12.90	[127.10–144.74]	Dense-fusion model; moderate instability across folds
Bi-GRU + attention	0.8476 ± 0.0228	[0.8227–0.8683]	0.0658 ± 0.0053	[0.0606–0.0712]	0.0160 ± 0.0026	[0.0136–0.0188]	72.33 ± 9.78	[65.06–84.61]	Bidirectional recurrent; highest fold-level variance

Metrics are reported as mean values with 95% percentile bootstrap confidence intervals.

Replicate counts: *n* = 150 for linear regression and random forest, *n* = 80 for XGBoost, *n* = 15 for Deep GRU, and *n* = 10 for transformer decoder, Bi-GRU+attention, and MBDFN.

The approach with the best predictive performance was Deep GRU, which yielded the highest *R*^2^ (0.8632) and the lowest MAE (0.0576), among all the approaches that were evaluated. This outcome shows the effectiveness of the recurrent sequence modelling in exploiting the temporal-style feature representation in case of explicit presence of temporal information in the input space. The second-highest performance (*R*^2^ = 0.8476) was obtained by Bi-GRU + Attention, which suggests that bidirectional contextual information and attention mechanisms can be used to gain extra modelling power, but with a wider uncertainty range, it means that the model is sensitive to data variation. The XGBoost model among the analysed models showed competitive results with *R*^2^ = 0.8240 and lowest complexity non-recurrent modelling strategy. The result illustrates that there is a significant amount of nonlinear information in the engineered temporal feature space that can be preserved without explicit recurrent memory.

Similarly, the transformer decoder achieved *R*^2^ = 0.8240, demonstrating that meaningful relationships can be captured from the structured temporal representation under the current dataset conditions by modelling using attention. The baseline models gave significantly poorer prediction performance Linear Regression had an *R*^2^ = 0.2226, confirming the strong non-linearity between the engineered descriptors and melody similarity. Random Forest showed a significant improvement over this linear baseline (*R*^2^ = 0.5893), demonstrating the usefulness of nonlinear ensemble methods over simple linear modelling for this task.

**Figure 3:**
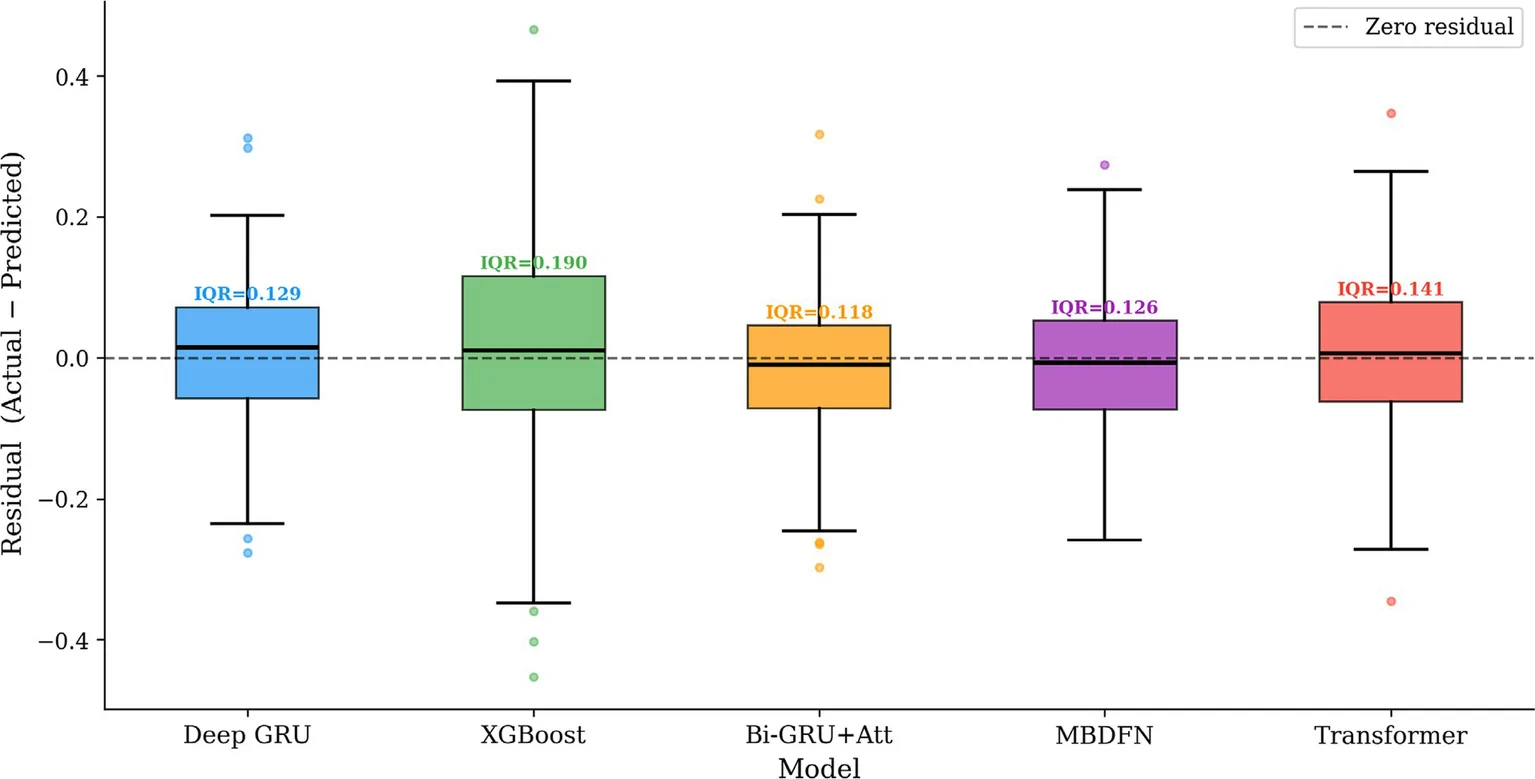
Residual boxplots for all compared models showing the distribution of prediction errors (actual – predicted). Boxes show median and interquartile range (IQR); whiskers extend to 1.5 × IQR; outliers shown as individual points.

All models compared are plotted in this Figure 3 to show the residual distributions. The boxplots in the residual plot show the actual distribution of the prediction errors, their median, interquartile ranges and the distribution of outliers. Models having a smaller residual distribution with closer median to zero have more consistent prediction behaviour. The residual distribution of Deep GRU and Bi-GRU + Attention models were relatively more concentrated, while the baseline models had a higher variability in errors.

**Figure 4:**
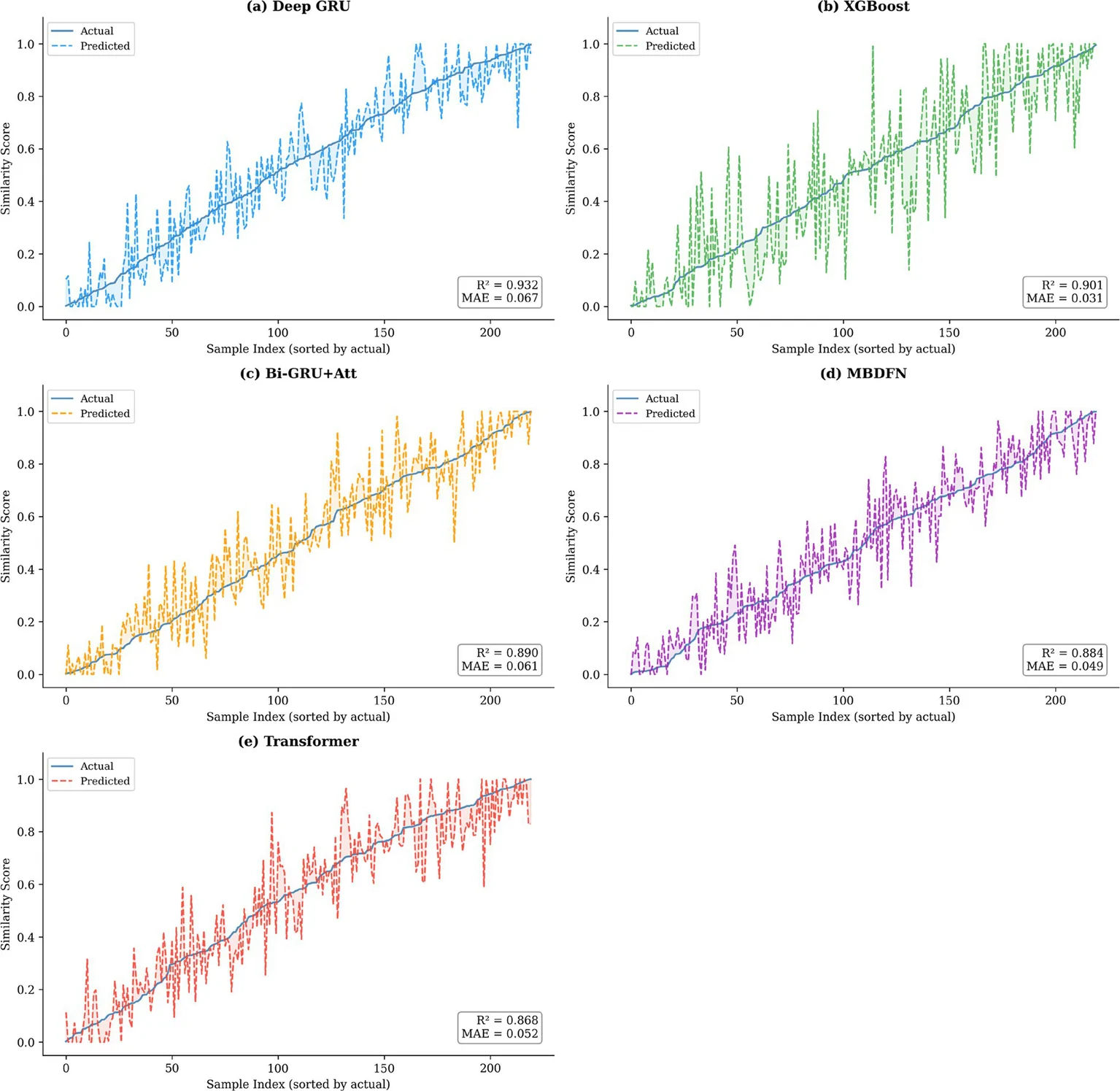
Actual-versus-predicted similarity scores for the compared models: **(a)** deep GRU, **(b)** XGBoost, **(c)** transformer decoder, **(d)** Bi-GRU + attention, and **(e)** Multi-branch dense fusion network.

Plots of actual versus predicted values of similarity as shown in Figure 4 further illustrate the agreement between similarity values predicted and observed. The closer the models are to the diagonal reference line, the better they predict and the less systematic error. The comparison indeed shows that nonlinear models are more capable of prediction than their linear counterparts (Pušica et al., 2025).

**Figure 5:**
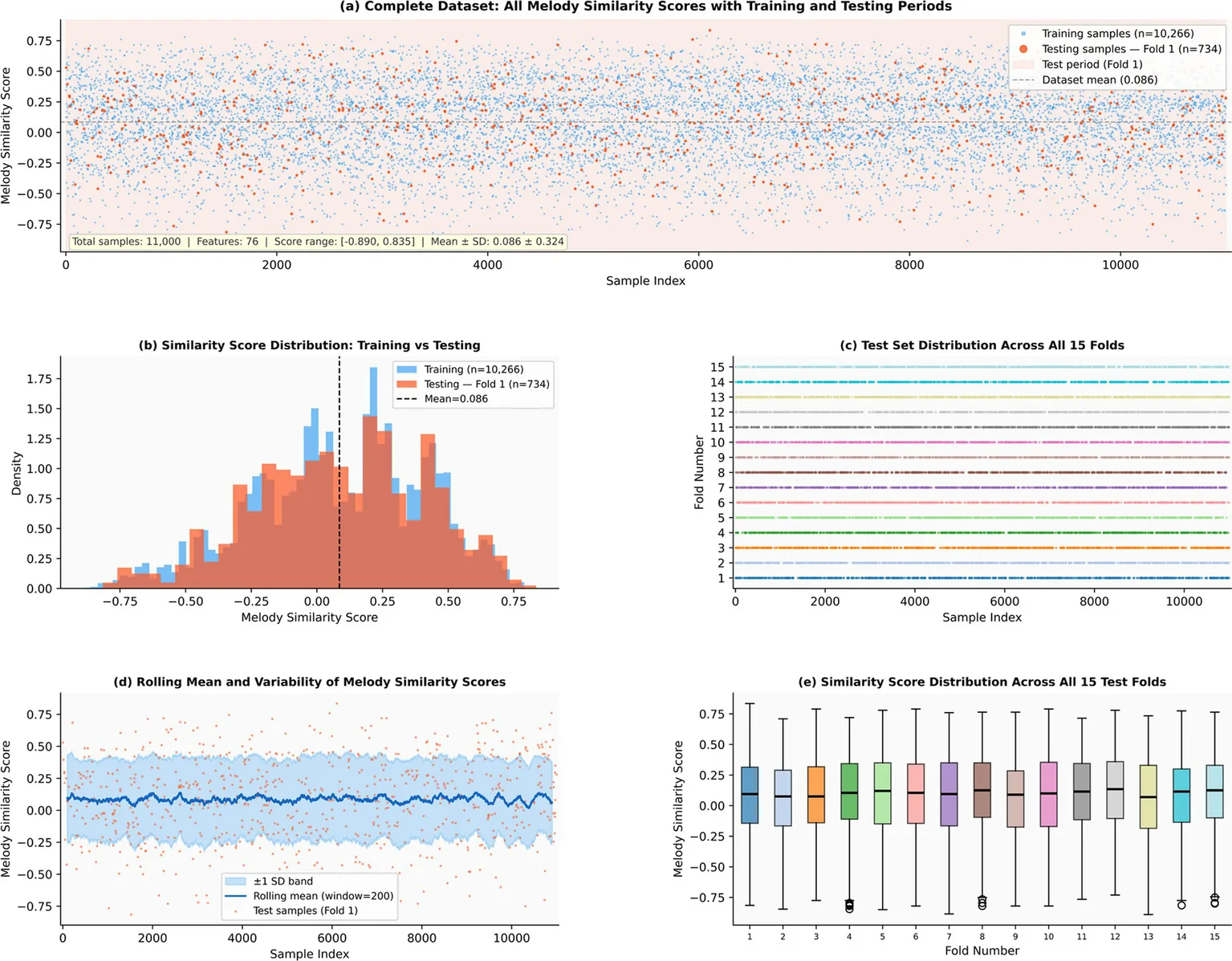
Complete dataset visualization with training and testing distributions. **(a)** Distribution of melody similarity scores for the complete dataset with training and testing samples; **(b)** comparison of similarity score distributions between training and testing datasets; **(c)** distribution of test samples across 15 cross-validation folds; **(d)** rolling mean and variability analysis of melody similarity scores; and **(e)** similarity score distribution across all test folds.

The distribution of the extended melody similarity dataset over the training and test sets is shown in Figure 5. The distribution across the partitions is relatively even, suggesting that the distribution across the data splits was not significantly skewed and lending credibility to the comparative analysis.

Results overall indicate that the explicit feature engineering for the temporal styles is useful and offers an intermediate representation layer appropriate for other model families. The results do not argue for a superiority of one architecture in general, but rather show that the performance of the model depends on the interplay between the temporal representation, model structure, and characteristics of the datasets. The statistical comparisons shown in Table 9 also show that the enhancements of the advanced models compared to the baseline models are statistically significant.

**Table 9:** Pairwise paired t-test results for model performance comparison based on 15-fold cross-validation *R*^2^ values (*df* = 14, *α* = 0.05).

Model	Mean Δ*R*^2^	*t*-statistic	*p*-value	Significance (*α* = 0.05)
A. deep learning/gradient-boosted models vs. baseline models				
XGBoost vs. linear regression	+0.613	91.00	<0.001	Significant
XGBoost vs. random forest	+0.236	112.51	<0.001	Significant
MBDFN vs. linear regression	+0.537	66.94	<0.001	Significant
MBDFN vs. random forest	+0.159	29.64	<0.001	Significant
Bi-GRU + attention vs. linear reg.	+0.475	51.42	<0.001	Significant
Bi-GRU + attention vs. random forest	+0.097	13.93	<0.001	Significant
Deep GRU vs. linear regression	+0.510	—	—	Significant
Deep GRU vs. random forest	+0.132	—	—	Significant
Transformer vs. linear reg.	+0.490	—	—	Significant
Transformer vs. random forest	+0.112	—	—	Significant
B. pairwise comparisons among deep learning/gradient-boosted models				
XGBoost vs. Bi-GRU + attention	+0.139	18.78	<0.001	Significant
XGBoost vs. MBDFN	+0.077	12.93	<0.001	Significant
MBDFN vs. Bi-GRU + attention	+0.062	11.94	<0.001	Significant
Random forest vs. linear regression	+0.377	58.03	<0.001	Significant

Mean Δ*R*^2^ represents the mean difference in fold-wise *R*^2^ values (positive = first model outperforms second). *t*-statistics and *p*-values from two-tailed paired t-tests on 15-fold *R*^2^ scores. All reported *p*-values < 0.001.

### 4.2 Effect of temporal-style feature engineering

To assess the effect of the proposed temporal-style feature representation, a representative ablation comparison was done using the Bi-GRU + Attention model under two feature conditions: static features alone and static features and temporal-style descriptors. The setting of static-feature involved the spectrogram descriptors and MFCCs and the pitch-contour features, MIDI-index features and embedding features. The temporally enriched setting included first-order deltas, second-order deltas, relative ratios, polynomial interactions, rolling means, and rolling variances. As it can be seen in Table 10, removing the temporal-style descriptors out of the Bi-GRU + Attention model reduced *R*^2^ from 0.890 to 0.801 (a reduction of 0.089), MAE increased from 0.0610 to 0.0856, and MSE increased from 0.0109 to 0.0197. This finding indicates that the suggested temporal-style descriptors provided valuable predictive data in this representative recurrent structure. Nevertheless, since not all model families had the complete static-feature baseline, this finding is construed as evidence based on ablation, as opposed to a full-fledged model-wise ablation comparison.

**Table 10:** Representative ablation evidence for the effect of temporal-style feature engineering.

Model/configuration	*R* ^2^	MAE	MSE	Interpretation
Bi-GRU + Attention with static features only	0.801	0.0856	0.0197	Performance decreased when temporal-style descriptors were removed
Bi-GRU + Attention with temporal-style features	0.890	0.0610	0.0109	Performance improved after adding temporal-style descriptors
Absolute change	+0.089	−0.0246	−0.0088	Temporal-style features provided a measurable improvement in this representative recurrent setting

Table 10 reports representative ablation values from a controlled single experimental run conducted on the original dataset (1,099 samples), where static and temporal feature groups are clearly separable by design. Confidence intervals for full model cross-validation performance are reported in Tables 8, 13. The extended dataset (11,000 samples) incorporates temporal-style features by construction across all 76 engineered variables, making component-level separation less meaningful in that setting.

### 4.3 Model behaviour, error diagnostics, and architectural comparison

Under the extended dataset conditions, the *R*^2^, MAE, and MSE of the recurrent architectures tested were evaluated, and Deep GRU had the highest predictive performance (Table 8). The results demonstrate that the temporally neatly encoded representation could be useful for recurrent modelling, as temporal dimension is not modeled purely from the sequential inputs. The results of the diagnostic analyses indicated that the prediction errors of Deep GRU exhibited relatively narrow residual distribution and no significant systematic biases. The comparison between actual and predicted values showed good agreement between the predicted and observed similarity values, and the loss curve of the optimization process showed stable convergence after the first optimization point. The combination of recurrent representation learning, temporal feature encoding and regularization strategies rendered a stable predictive framework under the current experimental conditions, as suggested by these behaviours.

**Figure 6:**
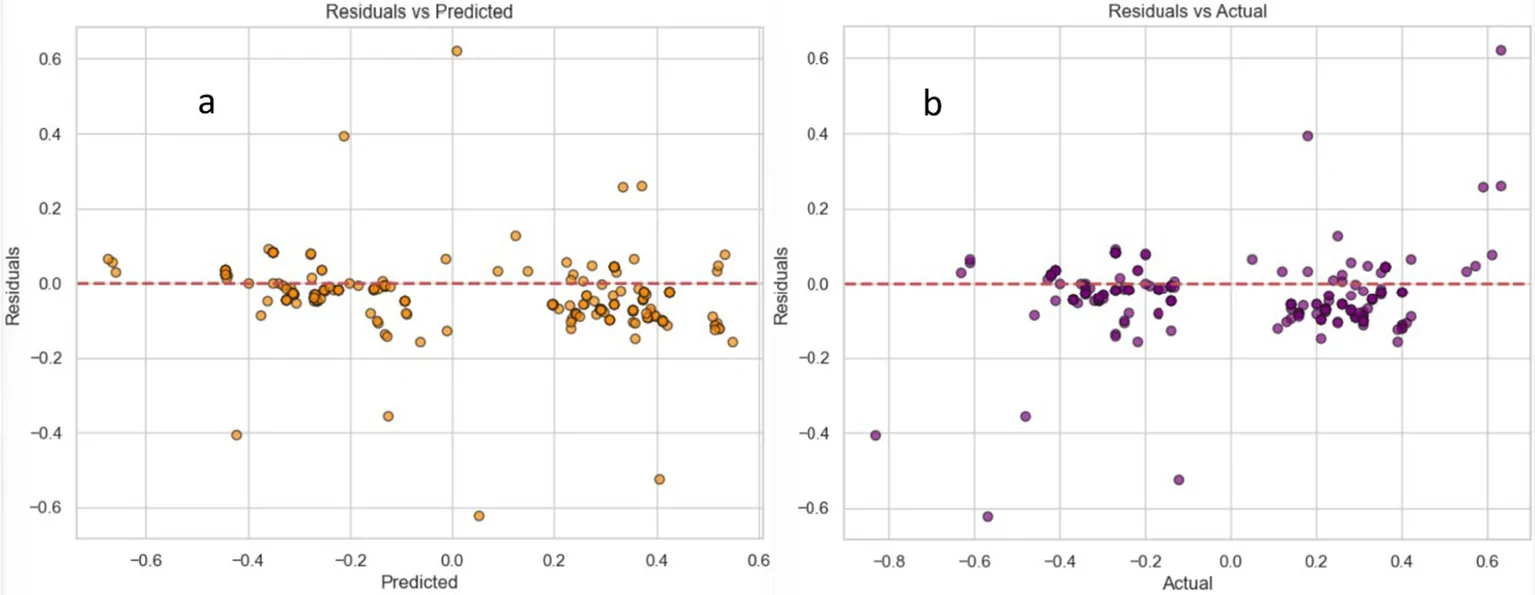
Diagnostic results for the deep GRU model **(a)** residuals versus predicted values **(b)** residual versus actual.

**Figure 7:**
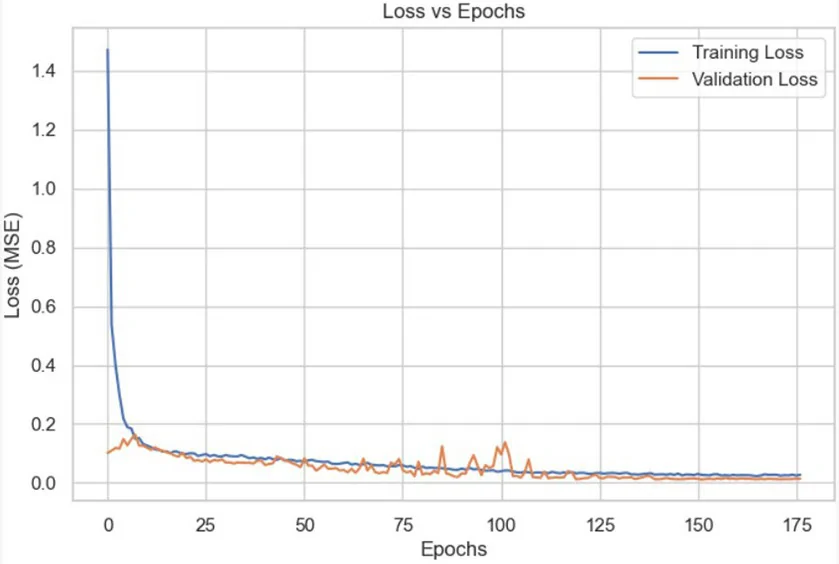
Loss vs. epoch performance of deep GRU.

The residual behaviour of the Deep GRU model is depicted in Figure 6, with the relationship between the prediction errors and the similarity values (predicted and observed) shown. The evolution of the training and validation losses is provided in Figure 7.

Ablation analysis was done on the original data set (Table 11) to examine the individual contribution of the architectural and feature components. Eliminating temporal descriptors (deltas, ratios and rolling statistics) lead to lower performance, thus providing a proof that these features were effective in prediction ability. Likewise, the removal of regularization terms or the reduction in the depth of recurrent networks negatively impacted performance, indicating that both feature representation and model configuration had an effect on the final prediction behaviour (Achuthan, 2025; Saeed and Omlin, 2023).

**Table 11:** Ablation study of the deep GRU model.

Configuration	*R* ^2^	MAE	MSE	Interpretation
Full model	0.932	0.067	0.011	Full temporal-feature and regularized GRU configuration
Remove deltas	0.886	0.070	0.013	Short-term temporal contrast becomes weaker
Remove ratios	0.892	0.069	0.0128	Proportional variation contributes modestly
Remove rolling means	0.900	0.068	0.0123	Local temporal stability contributes to prediction
Remove BatchNorm	0.895	0.071	0.013	Training stability is reduced
Remove Dropout	0.882	0.074	0.0136	Generalization decreases, suggesting overfitting
Shallower GRU	0.897	0.070	0.0127	Lower recurrent capacity reduces performance
Remove deeper dense head	0.889	0.071	0.013	Dense fusion contributes to nonlinear integration

Table 11 reports representative ablation values examining the contribution of individual architectural components of the Deep GRU model. These ablation experiments were conducted on the original dataset under a single controlled run to isolate the effect of each component. Confidence intervals for full Deep GRU model performance across 15 folds are reported in Tables 8, 13. Architectural ablation findings are expected to generalise across dataset scales as they reflect structural model properties rather than dataset-specific effects.

The ablation results should not be confused with the extended-dataset cross-validation results presented in Table 8, however, which are model comparisons with uncertainty estimates.

On the extended dataset (Table 8), the XGBoost model performed best in terms of predictions, with an *R*^2^ value of 0.8240, an MAE of 0.0913, and an MSE of 0.0185. XGBoost did not have the best overall *R*^2^ of any architecture, but out of the non-recurrent models, XGBoost had the greatest *R*^2^, suggesting that the temporal features explicitly engineered to the model contain a significant amount of non-linear information that is missed by recurrent memory mechanisms.

The performance of the prediction in terms of similarity to melody in the XGBoost models was significantly better than the prediction performance of the baseline models, suggesting the relationship between temporally engineered descriptors and melody similarity is very nonlinear. The temporal-style representation is informative structured variables that can be effectively learned by gradient-boosted decision trees and conventional ensemble learning methods due to its capability of modeling complex feature interactions. The residual analysis also revealed moderate prediction error concentration and small systematic bias, and the training and validation behaviour revealed moderate convergence stability in the regularization and early stopping applied. The results indicated that XGBoost offers a competitive trade-off between the predictive capability, computational efficiency, and interpretability of structured melody similarity modelling (Pesek et al., 2020).

**Figure 8:**
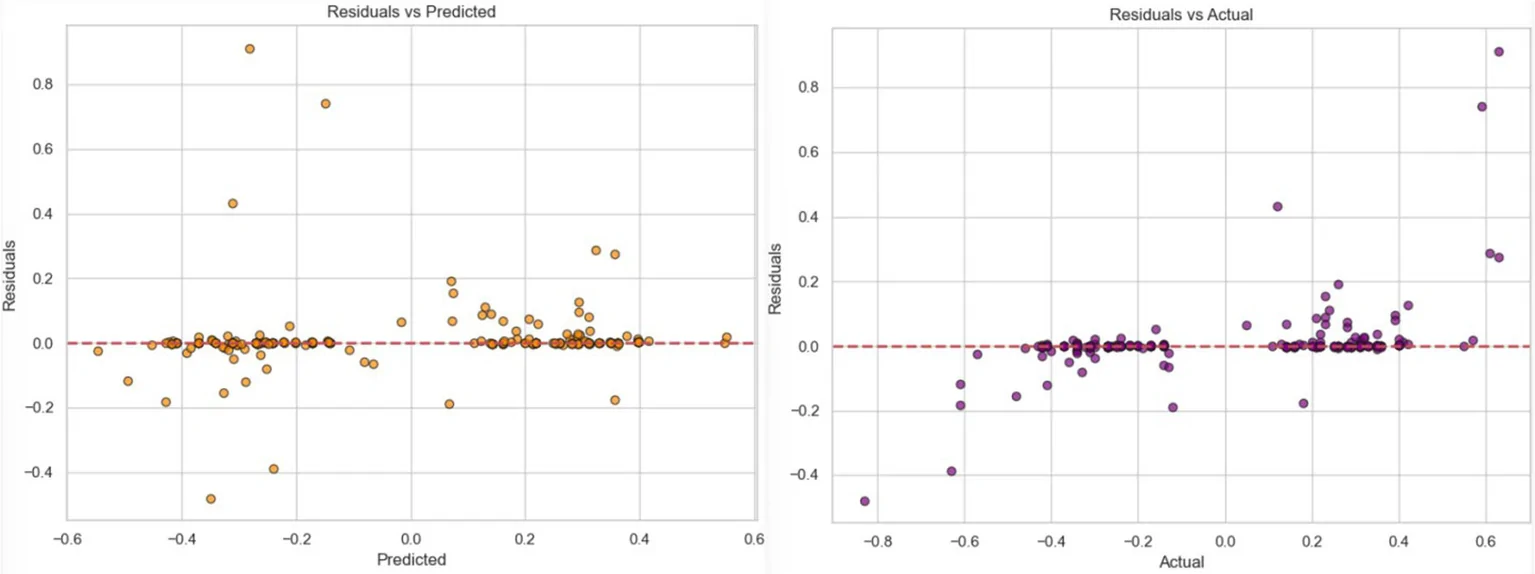
Diagnostic results for the XGBoost model residuals versus predicted values.

Figure 8 presents the residual behaviour of the XGBoost model, showing the distribution of prediction errors relative to predicted and observed similarity values.

**Figure 9:**
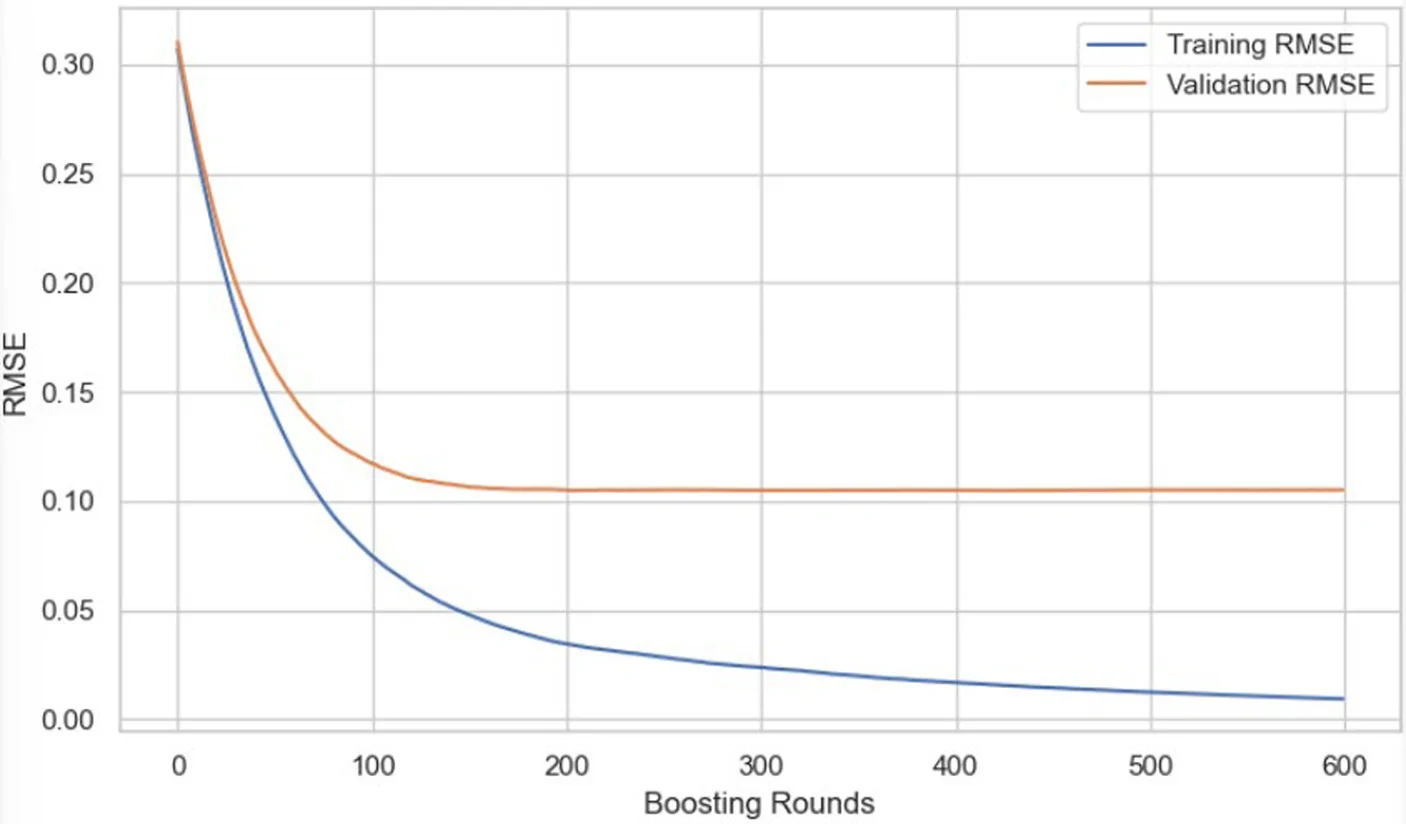
Training and validation performance of the XGBoost model.

The performance curves for training and validation (Figure 9) suggest that the optimization behaviour is stable and reveal little evidence of overfitting when regularized using the strategy adopted. For the XGBoost model, SHAP was used to explore the contribution of individual features further. The analysis showed that embedding-related variables, temporal delta descriptors and spectral ratio features were some of the influencing predictors of the model output. This result indicate that high-level latent representations as well as explicitly engineered temporal descriptors played a role in predicting melody similarity.

**Figure 10:**
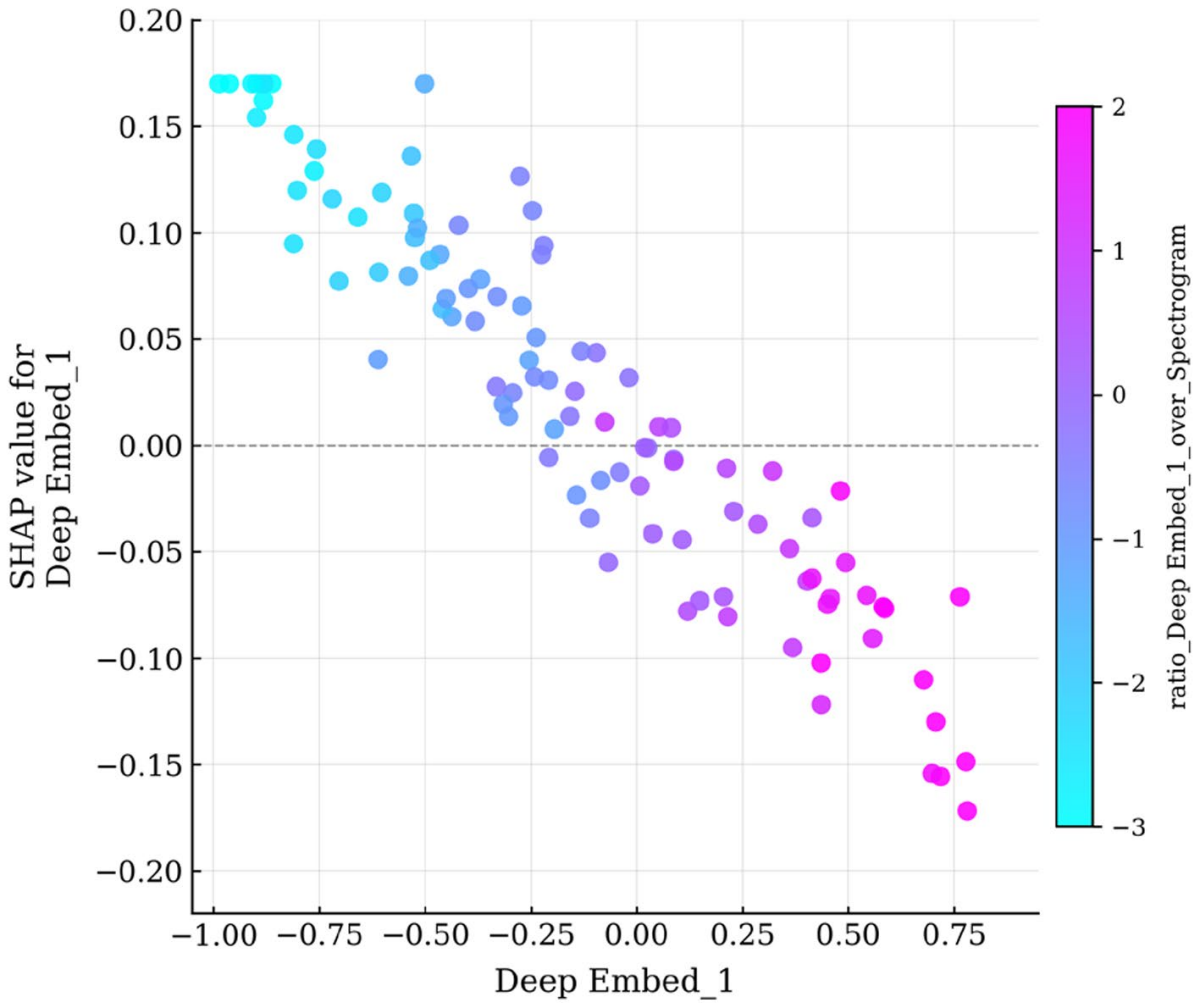
SHAP dependence plot showing the relationship between a selected embedding feature and XGBoost prediction output.

Figure 10 depicts SHAP dependence analysis provides a visualization of how important model features relate to the model’s prediction.

**Figure 11:**
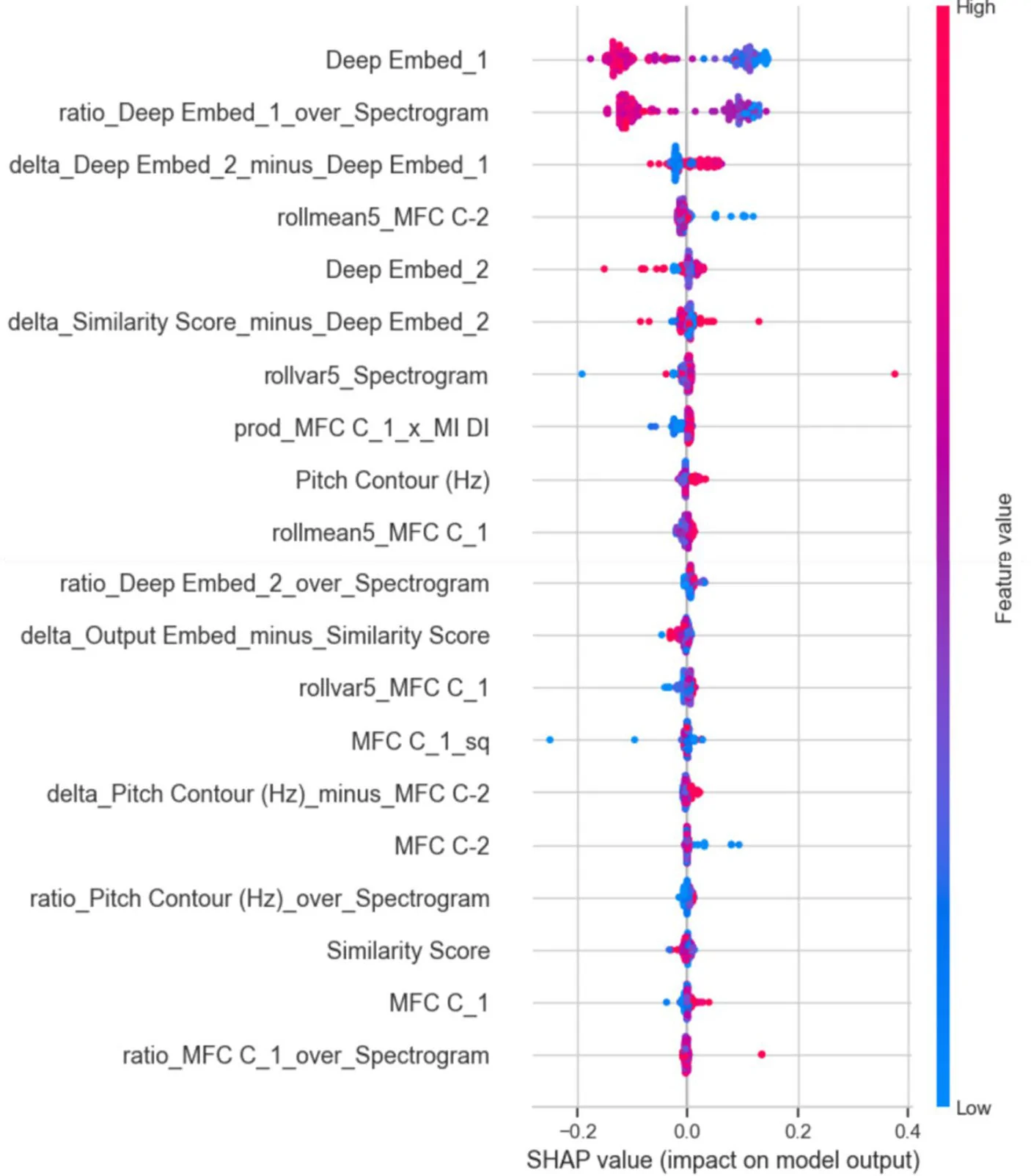
SHAP summary plot showing global feature importance for the XGBoost model.

The SHAP summary plot gives a summary of the relative importance of key variables over the data set. It is important to note that engineered temporal descriptors could be correlated, and, so, SHAP values link to the model’s explanations rather than to causal relationships between individual features and the human perception of melodic similarity (Figure 11).

The Transformer Decoder outperformed the current experimental conditions with competitive predictive performance, with an *R*^2^ of 0.8240, MAE of 0.0789 and MSE of 0.0185 (Table 8). This performance demonstrates the effectiveness of using the explicitly designed temporal feature representation and the ability to learn the nonlinear relationships between features in the structured melody similarity feature space by attention-based modelling. The Transformer Decoder was found to have significantly higher prediction accuracy than the baseline models, and demonstrated that meaningful patterns can be discovered in temporally enriched descriptors even when the raw sequential audio inputs are not directly provided to an attention model.

It was still, however, not as effective as the best recurrent models, such as Deep GRU, indicating that the effectiveness of attention-based architectures is highly dependent on model design, input representation, and dataset characteristics. The diagnostic analyses revealed stable training behaviour and convergence in the optimization process. The prediction behaviour of the residual distributions was consistent, but not as precise as the best-performing architectures. This could relate to the current structured tabular representation, the size of the dataset or limited architecture-specific optimization done in this study.

**Figure 12:**
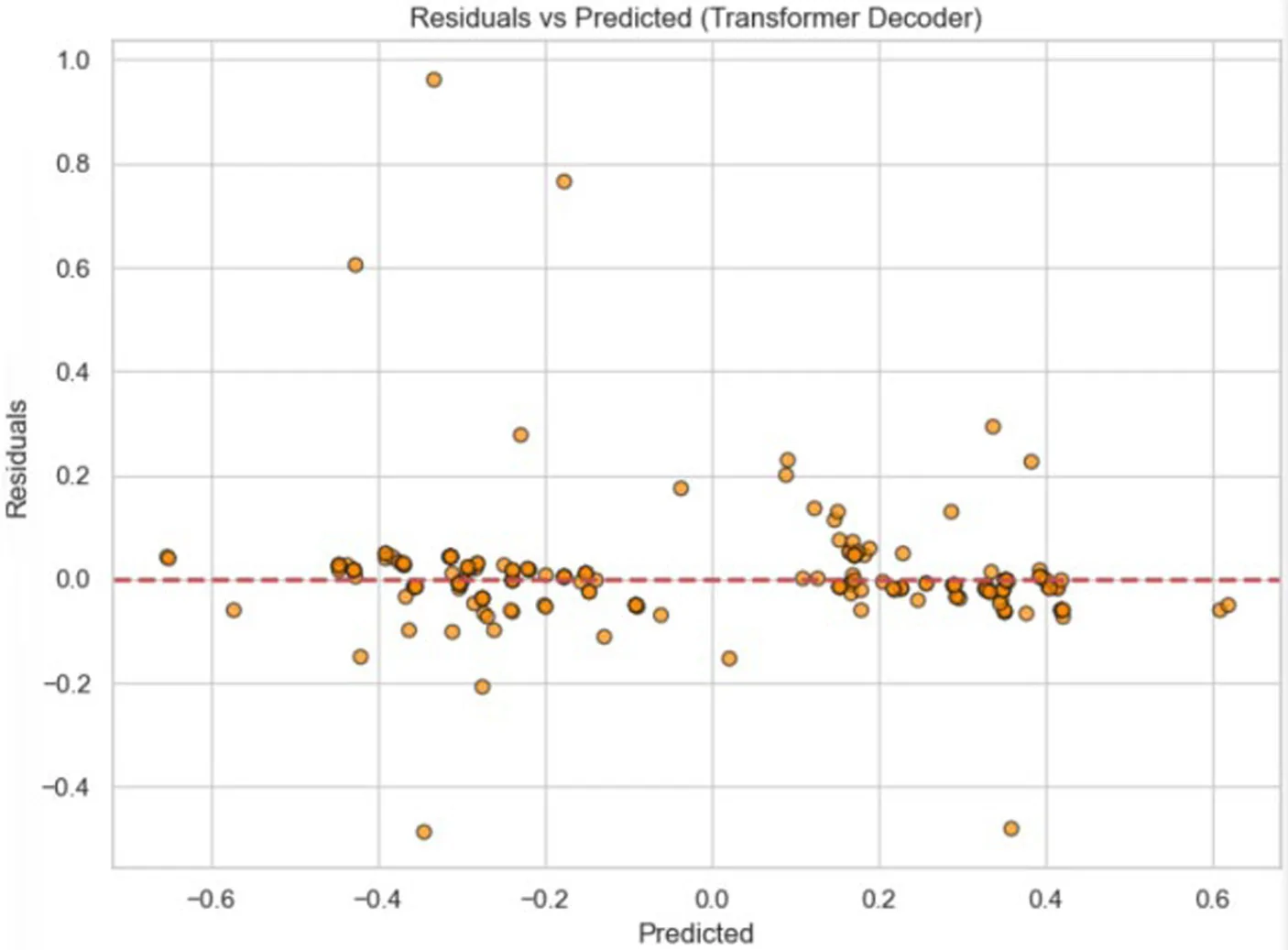
Diagnostic results for the transformer decoder residuals versus predicted values.

Hence, it should not be taken as a general drawback of Transformer architecture (Figure 12), as this model has been used successfully in more complex sequence modelling problems in larger model sizes but it is merely a statement concerning the current experimental setup.

**Figure 13:**
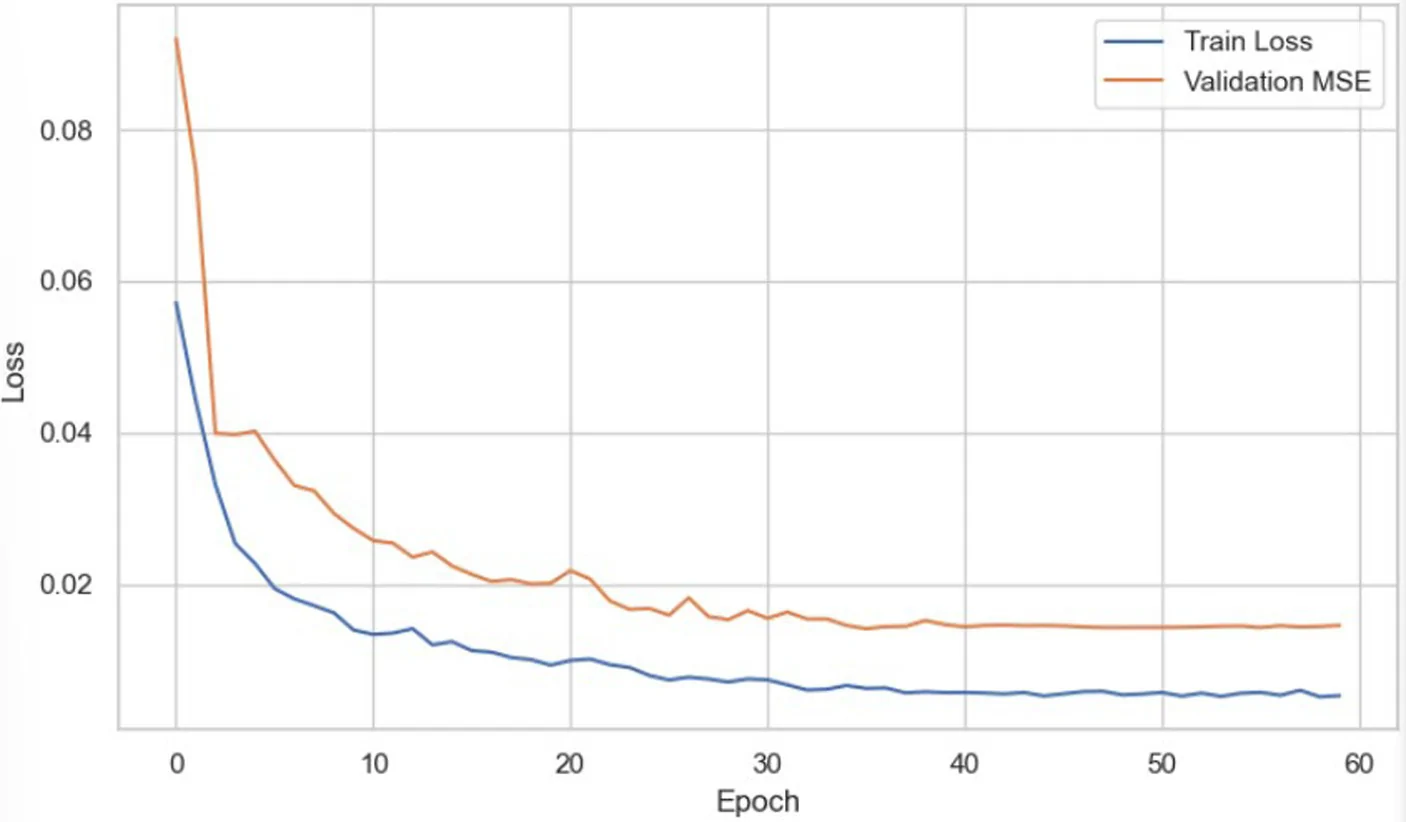
Training and validation loss curves for the transformer decoder.

The training and validation loss curves shown in Figure 13 demonstrate the convergence behaviour and stability of the optimization process.

**Figure 14:**
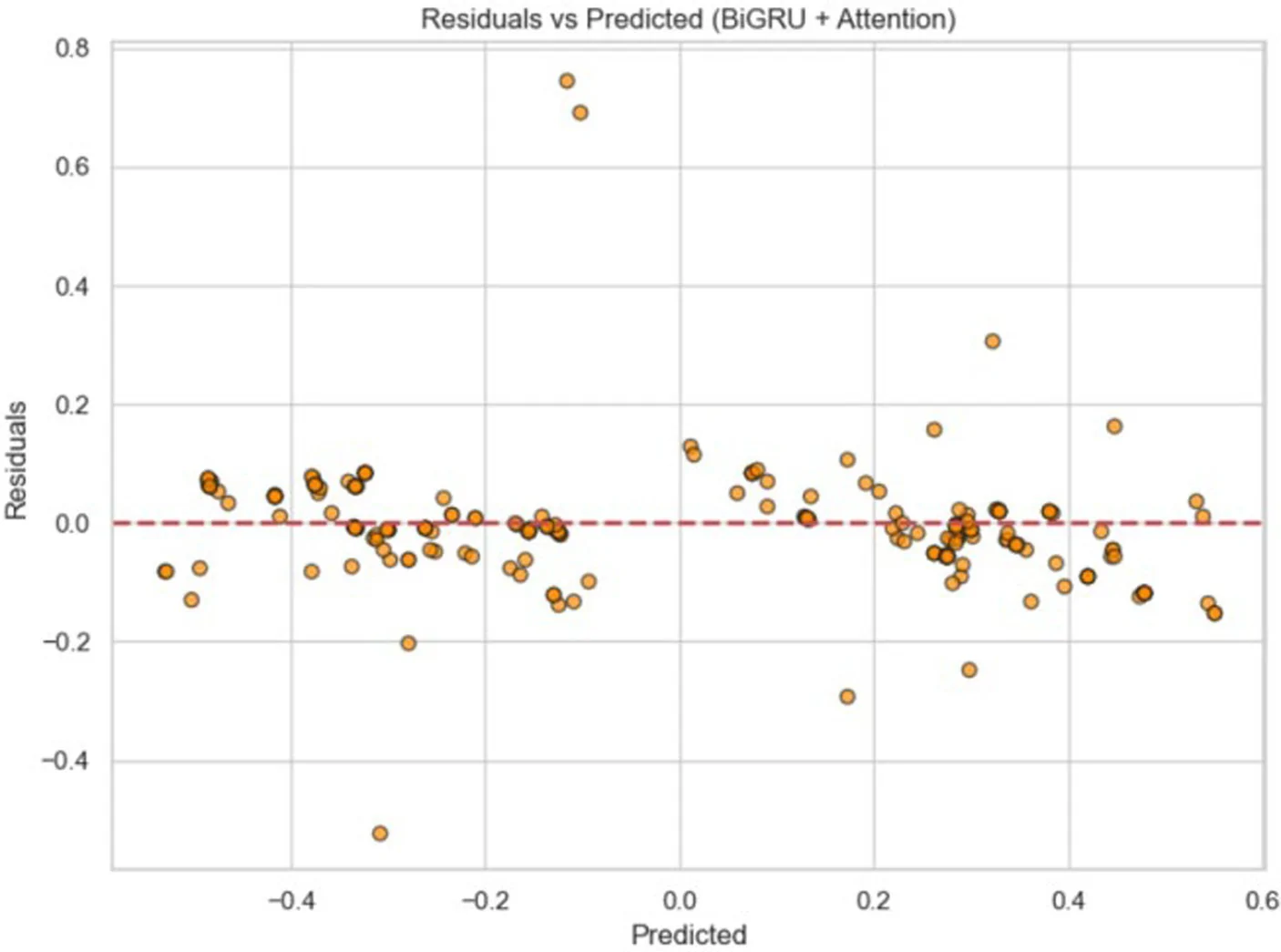
Actual-versus-predicted similarity scores for the Bi-GRU + feature-aware attention model.

The Bi-GRU + Feature-Aware Attention model demonstrated strong predictive performance on the extended dataset, achieving a mean *R*^2^ of 0.8476 (95% CI: 0.8227–0.8683), together with an MAE of 0.0658, an MSE of 0.0160, and a MAPE of 72.33%. These results ranked the model second among all evaluated architectures, behind the Deep GRU but ahead of the Transformer Decoder, XGBoost, and the baseline models. Although the bidirectional recurrent architecture with attention effectively exploited the engineered temporal-style feature representation, its slightly wider uncertainty range compared with the Deep GRU suggests greater sensitivity to variations in the evaluation data. The results indicate that combining bidirectional contextual learning with an attention mechanism can enhance predictive capability, although the additional architectural complexity did not translate into superior performance over the Deep GRU under the current experimental conditions. Figure 14 presents the agreement between predicted and observed melody similarity values for the Bi-GRU + Feature-Aware Attention model.

**Figure 15:**
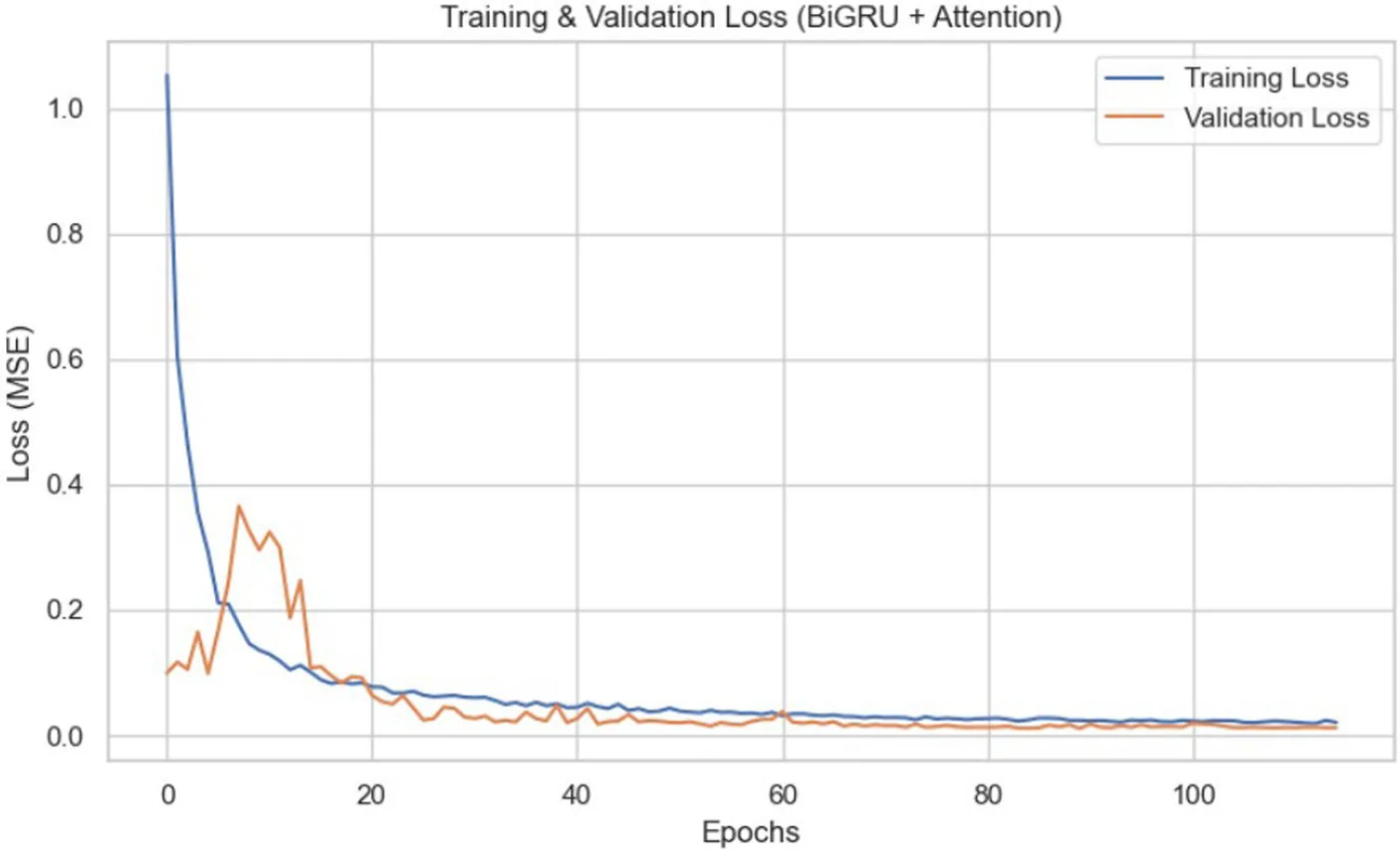
Diagnostic results for the Bi-GRU + feature-aware attention model.

Figure 15 shows the residual distribution behaviour, indicating prediction error variability and potential systematic bias.

**Figure 16:**
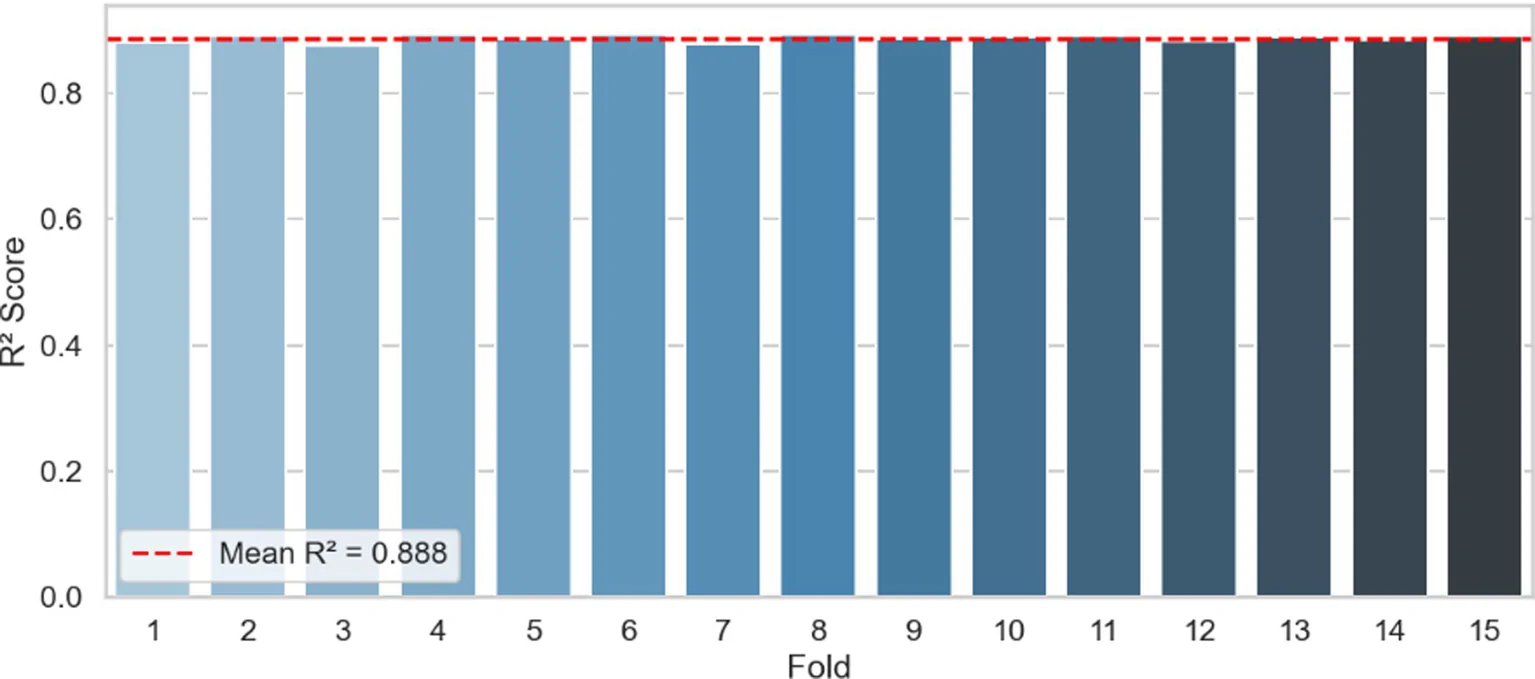
Training and validation loss curves for the Bi-GRU + feature-aware attention model.

Training and validation loss curves are shown in Figure 16, which show the convergence behaviour throughout the optimization of the model.

Ablation analysis was conducted to evaluate the contribution of individual architectural components and temporal-style descriptors, as summarized in Table 12. The greatest loss of model performance occurred after the removal of temporal feature engineering (Variant A2, Δ*R*^2^ = −0.089), suggesting that the explicit temporal descriptors had a significant impact on model performance. The results indicated that removing attention, bidirectional recurrent processing, or regularization components also led to significant performance decreases, indicating that feature representation as well as architectural design were responsible for reducing predictive ability.

**Table 12:** Ablation study of the Bi-GRU + feature-aware attention model.

Variant	Modification	*R* ^2^	MAE	MSE	Interpretation
Full model	Bi-GRU + Attention + temporal features + Dropout + BatchNorm	0.8901	0.0610	0.0109	Full model configuration
A1	Removed attention layer	0.8427	0.0723	0.0158	Attention contributed to performance
A2	Removed feature engineering	0.8014	0.0856	0.0197	Temporal-style features contributed substantially
A3	Replaced Bi-GRU with single-direction GRU	0.8279	0.0761	0.0173	Bidirectional context contributed to prediction
A4	Removed Dropout and BatchNorm	0.8142	0.0812	0.0189	Regularization improved stability
A5	Reduced GRU layers from 3 to 1	0.8235	0.0784	0.0178	Reduced depth weakened representation capacity
A6	Removed polynomial and rolling features	0.8356	0.0742	0.0165	Contextual temporal features contributed modestly

Table 12 reports representative ablation values for Bi-GRU + Attention variants A1–A6, conducted on the original dataset under a single controlled run. Each variant isolates the contribution of one architectural or feature-level component. Confidence intervals for the full Bi-GRU + Attention model across 15 folds are reported in Tables 8, 13. These findings are interpreted as indicative evidence of component contributions rather than definitive cross-validated estimate.

The ablation results are to be understood as evidence on the level of the individual components, not as uncertainty estimates for the entire extended data set. The main model comparison should thus be based on evaluation results obtained from the bootstrap shown in Table 8.

For the extended dataset (Table 8), the Multi-Branch Dense Fusion Network (MBDFN) performed well with *R*^2^ reaching 0.6601, MAE 0.1386, and MSE 0.0357. While its predictive power was not as high as the best recurrent and gradient-boosted models, MBDFN was capable of using structured feature interactions by processing them separately via feature-groups and/or applying nonlinear dense fusion. Explicitly organized feature representations are found to be useful for predicting information even without an explicit recurrent or attention mechanism, as can be seen in the performance of MBDFN.

The other possibility is that the uncertainty range is larger than in some models, in this case SD = 0.0355 for *R*^2^, which indicates that the uncertainty of the data will be larger and that the generalization stability with respect to different samples of the bootstrap will be less. The residual analysis revealed mostly centered prediction errors and the training and validation curves demonstrated stable optimization behaviour.

**Figure 17:**
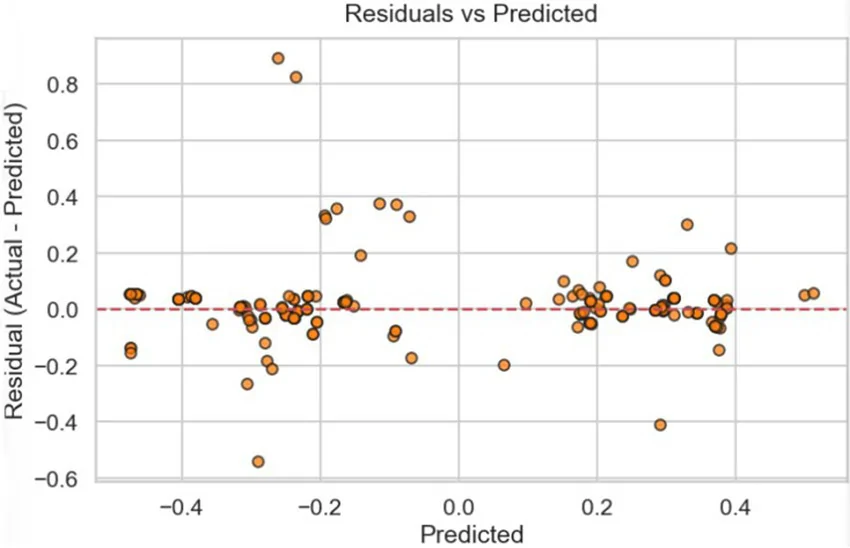
Actual-versus-predicted similarity scores for the multi-branch dense fusion network.

**Figure 18:**
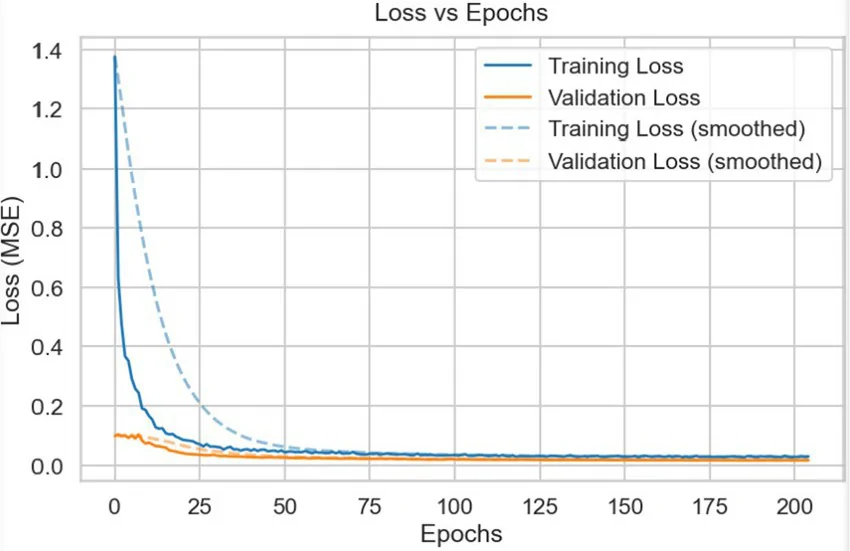
Diagnostic results for the multi-branch dense fusion network.

The results indicate that dense feature fusion can be used as a complementary modelling approach in the case of temporally engineered representations, but its usefulness is very sensitive to the structure of the features and the characteristics of the data sets (Figure 17). This plot displays the actual versus predicted similarity values for the MBDFN model, demonstrating the level of agreement between the outputs of the model and the data when using the MBDFN model. The residual behavior of the MBDFN model is presented in Figure 18, which also displays the distribution of errors and the possible bias in the model predictions. It should be noted that MBDFN is not a two dimensional spectrogram-based convolutional model, but a multi-branch dense fusion model, which is based on the engineered feature groups. Thus, it should be used as part of structured temporal feature representation, not image-based music modelling.

For each of the seven architectures, a definite order of performance is established. The temporal-style features gave good fits in recurrent modelling, with deep GRU (*R*^2^ = 0.863) giving the strongest and most stable fit. A second tier was composed of Bi-GRU + Attention (*R*^2^ = 0.848), Transformer Decoder (*R*^2^ = 0.824) and XGBoost (*R*^2^ = 0.824), similar in accuracy but with larger bootstrap uncertainty than Deep GRU, and XGBoost being really competitive without requiring any sequential structure. The sequence-aware models were not only significantly better than MBDFN (0.660), but also significantly better than the feature-based grouping models, such as the Random Forest (0.589) and Linear Regression (0.223) models, which verified the task’s strong nonlinearity. In general, the best trade-offs of accuracy and stability are achieved with Deep GRU and XGBoost, while Attn and Fusion models sacrifice stability for flexibility in architecture.

### 4.4 Cross-validation stability of all compared models

This section examines the predictive skill of all compared models using 15-fold cross-validation, separately from the bootstrap-based uncertainty estimates reported in Table 8. The aim of the current analysis is to compare model behaviour and stability across cross-validated folds, not to re-assess predictive accuracy. Overall, the fold-wise results showed that performance of the models was consistent across various data partitions indicating that the reported results were not dependent on a particular favourable train-test split (Table 13). The advanced nonlinear models tended to be more accurate, and exhibited a more robust generalization property on repeated evaluations, than the baseline models. XGBoost had the best performance among the non-recurrent models and Deep GRU had the best performance among the recurrent models with relatively low standard deviation across the cross-validation folds. Despite achieving competitive prediction performance, the Bi-GRU + Feature-Aware Attention model showed the highest fold-to-fold variation, indicating that the model is more sensitive to the partitioning of the data and the initialisation. The Transformer Decoder and MBDFN also achieved an intermediate performance, with their prediction errors being between the best models and the simple ones. The prediction error of Linear Regression was highest and it was the least generalizable baseline models.

**Table 13:** Fifteen-fold cross-validation stability of recurrent models.

Model	Relative stability	Fold-to-fold variability	Overall observation
Linear regression	Low	High	Weak baseline with poor generalization
Random forest	Moderate	Moderate	Improved over linear regression but below advanced models
XGBoost	High	Low	Most stable non-recurrent model
Deep GRU	High	Low	Most stable recurrent model
Transformer decoder	Moderate	Moderate	Stable but sensitive to dataset characteristics
MBDFN	Moderate	Moderate	Competitive but showed moderate variability
Bi-GRU + attention	Moderate	Highest among advanced models	Competitive accuracy with greater sensitivity to data partitioning

**Figure 19:**
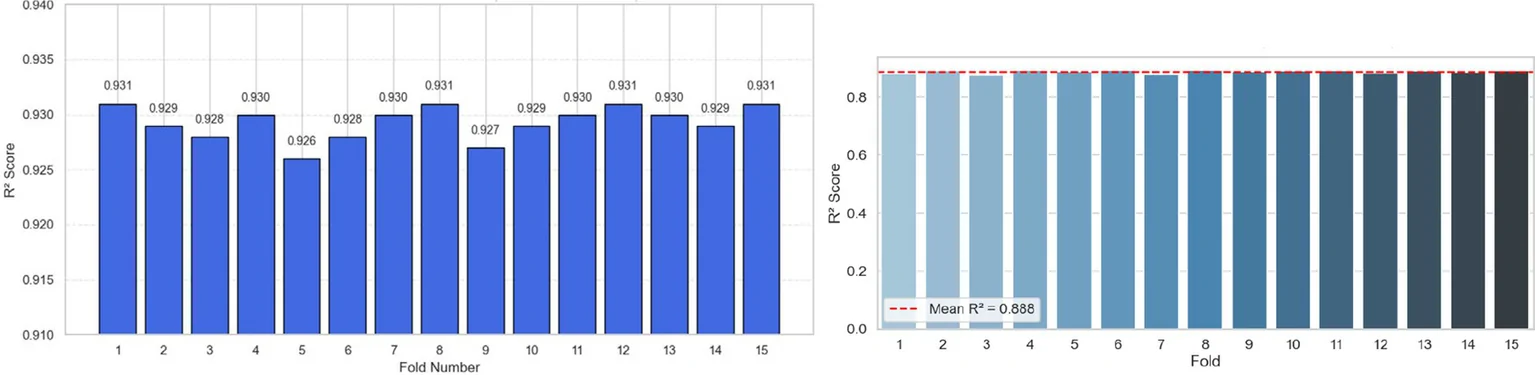
Fifteen-fold cross-validation *R*^2^ distributions for the recurrent models, including Deep GRU and Bi-GRU + attention.

Linear Regression had the highest prediction error and was the least well generalizing of the baseline methods. Random Forest did much better than the linear baseline, but not as well as the more sophisticated deep learning and gradient-boosting models. Overall, the cross-validation analysis confirms the robustness of the proposed temporal-style feature engineering framework by showing that the rankings of the models observed are stable with respect to the repeated partitions of the data and not due to a single lucky split. The *R*^2^ distributions by folds are plotted for all models compared in Figure 19.

### 4.5 Interpretability and musicological discussion

The proposed temporal-style feature engineering framework consistently boosted the performances of melody similarity prediction for all the tested machine learning and deep learning models. Overall, the advanced models significantly outperformed the baseline models without explicit temporal descriptors, showing that temporal descriptors that are explicitly engineered can effectively capture sequential musical characteristics. Deep GRU showed the best predictive performance of all the models evaluated, suggesting that gated recurrent learning is the best suited technique to model temporal dependencies in the engineered feature space. Bi-GRU + Feature-Aware Attention was the second method, and XGBoost was the best non-recurrent model that was both accurate and interpretable (using SHAP) (Merritt et al., 2023; Mobin et al., 2025). The Transformer Decoder and Multi-Branch Dense Fusion Network (MBDFN) also yielded competitive results, but was more sensitive to feature representation and model configuration. The musicological interpretation of the most influential temporal descriptors identified by SHAP analysis and permutation importance is summarized in Table 14.

**Table 14:** Musicological interpretation of influential temporal descriptors.

Feature group	Model-level meaning	Musicological interpretation	Caution
Pitch deltas	Short-term pitch movement	Melodic contour, interval direction, and phrase movement	Does not fully capture melodic meaning alone
Second-order pitch deltas	Change in pitch movement	Curvature, acceleration, or expressive transition	Sensitive to noise and segmentation
Spectral ratios	Proportional spectral change	Timbre, dynamic contrast, and texture fluctuation	May reflect recording or production variation
Rolling mean	Local temporal stability	Sustained melodic or timbral pattern	Depends on window length
Rolling variance	Local temporal variability	Ornamentation, phrase-level variation, and expressive fluctuation	May vary by genre and instrumentation
Embedding deltas	Change in latent representation	High-level shift in musical/acoustic structure	Should be interpreted cautiously because embeddings are latent

The interpretability analysis revealed that descriptors derived from embeddings, temporal pitch variations, spectral ratios, and rolling statistical features always contributed to predicting melody similarity. The pitch related descriptors mainly described melodic contour and development of phrases, while the spectral ratios and rolling statistics focused on changes in timbre, articulation, and temporal stability. Embedding based descriptors reflected high level latent musical information and thus should not be interpreted unconditionally as the musical characteristics, as they do not directly correspond with the observable music features. In conclusion, the results confirm the hypothesis that temporal-style feature engineering has a positive impact on performance and interpretability of the model (Zhao et al., 2025; Xiong et al., 2025).

However, SHAP values and permutation importance only describe model responses, not human perception of musical similarity and future work should follow-up these findings with listener-based similarity measures and musicological expert annotations. Two-tailed paired t-tests were conducted between models to further investigate if differences observed between models were statistically significant based on the results of the fold-wise cross-validation. Table 9 summarizes the results.

The statistical comparisons validated the significance of the performance improvements achieved by all of the advanced machine learning and deep learning models over the baseline models, which were generally conventional (*p* < 0.001), meaning that the improvements were not likely to be due to random variation across the data partitions. Together, the bootstrap evaluation, the cross-validation analysis, the ablation experiments, the feature attribution analysis and the statistical significance testing give complementary evidence of the robustness of the proposed modelling framework. The results are limited to the specific set of data and experimental setup, but the proposed temporal-style feature engineering framework offers a good starting point for further melody similarity prediction research with larger datasets, artist independent evaluation protocols and perceptually validated similarity annotations.

## 5 Scientific significance and methodological insight

The work is a contribution to the modeling of melody similarity by reframing the problem of modeling temporal representation as a feature-level design problem in addition to an architectural modeling problem. Time structure is often addressed with recurrent, convolutional or attention-based networks in much of the literature on music information retrieval. Although these techniques are still considered significant, the current research questions the possibility of making temporal information explicit as well, through structured feature transformations, such as deltas, relative ratios, interactions of polynomials, and rolling statistical descriptors (Huang et al., 2025).

The key methodological learning is that temporal-style descriptors are able to offer a shared representation tier between various model families. By enabling short-term variation, proportional change, and local stability to be compared within the same feature space, the framework enables recurrent models, tree-based ensembles, dense-fusion models, and attention-based models to be compared under more consistent conditions of input. This does not mean that time descriptors that are engineered take the place of sequence models. Instead, the findings indicate that explicit temporal representation could alleviate part of the load that is imposed on model architecture, particularly when data are limited. This point of view applies to the analysis of melody similarity since over time, melodic perception is determined not just by the static acoustic or embedding value but also by the dynamic variation of pitch, timbre, energy and local phrase structure. The given framework has offered a more inspectable method of reflecting these changes, yet have retained a flexible model choice. More broadly, the same principle may be useful in other temporally structured tasks where datasets are moderate in size and interpretability is important, such as acoustic monitoring, biomedical signal analysis, sensor-based modeling, and human-activity recognition. But these extensions must be separately justified, which cannot be supposed solely on the basis of the current results.

## 6 Applications and practical deployment

The proposed temporal-style feature representation can be applicable in music information retrieval systems where data availability, computational resources, and interpretability is practical. Since the framework does not require end-to-end training of raw audio, instead relying on pre-extracted structured descriptors, it can be used in workflows where raw audio processing is costly and requires licensing or is hard to reproduce. Similarity estimation is useful in music recommendation and similarity-search systems, where it is used to generate playlists, conduct content-based searches and organize music collections.

Explicit temporal descriptors can be used to avoid such systems using only static descriptors. Lightweight models like XGBoost can be used when cheaper prediction and interpretation are necessary based on the application requirement, and when sequential dependencies are of importance and offline computation is acceptable (Cao et al., 2025). It can also be used to explainable music analytics with the help of the framework. With feature-attribution techniques, temporal descriptors, including pitch deltas, spectral ratios, rolling means and rolling variances may deliver interpretable signals as to melodic contour, timbral change, local stability, and expressive variation. These descriptions are to be considered model-level interpretations and not as direct evidence of the listener perception. These explanations would require further validation by a listener study, symbolic annotation, or analysis reviewed by a musicologist, before being used to explain to humans, in a human-facing recommendation system or educational system.

## 7 Reproducibility and practical constraints

The framework was created keeping in mind reproducibility and experimental control. The study eliminates the reliance of raw-audio preprocessing options as well as permits different model families to be evaluated using a standard feature matrix. These were the same key evaluation measures, feature preprocessing logic, and hold-out testing strategy, which were used throughout the compared models. Where applicable, regularization, early-stopping and learning-rate scheduling have also been applied to reduce overfitting and achieve greater stability of training. Meanwhile, there are a number of practical limitations that one must take into consideration. The dataset was built out of processed descriptors based on publicly available sources of music-information, and the ultimate results are conditional on the quality, compatibility, and representativeness of the descriptors. In the current revision, fold-wise mean ± SD and 95% confidence intervals are reported for all four metrics across all seven compared model families under identical 15-fold cross-validation conditions, providing complete uncertainty quantification for every reported result (Ouyang et al., 2025).

Consequently, the results of the cross-validation are to be interpreted as having evidence of recurrent-model stability and not as a full-scale statistical comparison of all archetypes. The limitation is that the similarity target was built via embedding-based cosine similarity as opposed to a human annotation of melodic similarity. This renders the target reproducible and open to controlled modeling, although it may not fully reflect or capture similarity perceived by the listener, cultural context or musicological nuance. Future research ought to confirm the framework with song-independent and artist-independent splits, larger and more diverse corpora, similarity labels based on listeners, and fully matched hyperparameter-search protocols across all models.

## 8 Conclusion

To account for the sequential nature of music, this study introduced a temporal-style feature engineering process involving temporal deltas, proportional ratios, polynomial interaction, and rolling statistical descriptors to predict melody similarity. The proposed model-agnostic representation allowed the comparison of a wide variety of machine learning and deep learning models, with an expanded dataset of 11,000 samples and 76 engineered features. The experimental results showed that the proposed framework achieved better predictive performance in every experiment compared to the traditional baseline models. The best overall predictive performance was obtained by the Deep GRU approach, and the second best was Bi-GRU + Feature-Aware Attention, thus providing high-level evidence of the benefit of recurrent architectures for modelling temporal musical relationships. XGBoost outperformed all other non-recurrent models in both terms of prediction accuracy and interpretation through feature attribution using SHAP values. The evaluation through bootstrapping, cross-validation analysis, ablation analysis, and statistical testing further validated the robustness and reliability of the proposed framework. The interpretability analysis also demonstrated that the descriptors derived from the embedding model and the temporal pitch variation, spectral ratios, and rolling statistics were significant in predicting melody similarity, further highlighting the power of explicit temporal-style feature engineering. While the results are promising, the proposed framework should be further validated in larger and more diverse music collections, similarity annotations generated by listeners, artist-independent evaluation protocols, and standard hyperparameter optimization to further confirm the generalizability and usefulness of the proposed framework.

## 9 Limitations and future work

The given framework was aimed at facilitating methodological clarity, interpretability and controlled comparison. Nevertheless, a number of limitations must be considered and these give guidelines on which future studies can be made.

- The dataset was built based on processed descriptors based on publicly available music-information sources. Though this was supportive to controlled comparison and reproducibility, the results might not necessarily be fully applicable across different genres, instruments, recording conditions, and musical cultures. The framework should be tested on bigger and more diverse music corpora in future.
- The similarity target was created by the embedding-based cosine similarity instead of by human annotation of similarity between melodies. This gave a continuous target which is reproducible but may not be fully representative of the similarity perceived by the listeners, cultural background or musicological subtlety. Comparisons between embedding-based similarity labels and the listener judgments, symbolic annotations of the melody, or the similarity evaluations reviewed by experts should be compared in the future work.
- Though all the models were tested with the same representation of main features and metrics, the comparison was not completely on the basis of architecture, number of parameters, and sensitivity to tuning. Future research ought to incorporate architecture-appropriate baselines, complete static-feature baselines of all models, reportable trainable parameters, and consistent hyperparameter-search protocols.
- Prior versions of this manuscript had a limitation concerning the setting of 15-fold cross validation, which is now corrected as fold-wise cross-validation result including mean ± SD and 95% confidence intervals are reported for all seven model families being compared. Fold level R^2^ values were compared by pairwise paired t-tests when they were available and by providing inferential evidence using non-overlapping confidence intervals for the other models. In the future, those data should be achieved for all models under fully matched hyperparameter search protocol to fully and fairly statistically compare them.
- SHAP and permutation-based analyses were instrumental in model checking, but they do not give any direct causal explanations of perceived melody similarity. Temporal descriptors that are engineered can also be correlated, which can have an impact on the stability of attribution. Future research ought to encompass feature-correlation diagnostics, uncertainty analysis of feature importance, attention visualization, and listener- or musicologist-informed validation of interpretability claims.
- It was found that the Deep GRU performed best in the experimental environment at hand, yet recurrent models might need more computing resources than lightweight ensemble models. Future research must consider effective recurrent variants, model compression, streaming compatible designs, and deployment time analysis of real world music retrieval and recommendation systems.

## Data Availability

The raw data supporting the conclusions of this article will be made available by the authors, without undue reservation.
