# Supplementary material for: Explicit encoding of temporal dynamics for interpretable melody similarity modeling: insight into machine learning models
Source: Front Comput Neurosci. 2026 Aug 12;20:1773922. doi: 10.3389/fncom.2026.1773922 (PMC13506812; doi:10.3389/fncom.2026.1773922)
Supplement: Supplementary file 1 [file Supplementary_file_1.docx]

**Appendix A**

**Table A1:** Tabular Explanation of Deep GRU

| Section | Code Block | Purpose |
| --- | --- | --- |
| Feature Engineering | Deltas, ratios, squares, products, rolling means | Create richer temporal-style features for GRU |
| Final Feature Set | engineered_cols + X_full | Combine base + engineered features |
| Normalization | MinMaxScaler() | Scale features to 0–1 range for stable GRU training |
| Sequence Shaping | X_scaled.reshape(-1, seq_len, 1) | Convert each sample into a sequence (timesteps × 1 feature) |
| Train/Test Split | train_test_split(...) | Split into training and testing sets |
| Deep GRU Model | Stacked GRUs (256→256→128) + Dense layers | Build deep recurrent model with dropout + batchnorm |
| Optimizer & Compile | AdamW + loss='mse' | Use AdamW with weight decay for generalization |
| Callbacks | EarlyStopping, ReduceLROnPlateau | Prevent overfitting and adapt learning rate |
| Training | model.fit(...) | Train GRU with validation monitoring |

**Table A2:** Important techniques in XGBoost

| Technique | Purpose | Key Effect |
| --- | --- | --- |
| Gradient Boosting | Build trees sequentially, each correcting error of the previous | Improves accuracy iteratively |
| Regularization (reg_alpha, reg_lambda) | Adds L1/L2 penalties | Prevents overfitting, stabilizes model |
| Shrinkage (learning_rate) | Scales contribution of each tree | Allows deeper boosting with better generalization |
| Subsampling (subsample, colsample_bytree) | Uses random subsets of rows/columns | Reduces variance, speeds training |
| Tree Pruning (max_depth, gamma) | Limits tree growth and enforce split thresholds | Controls complexity, avoids overfitting |
| Handling Missing Values | Learns default directions for missing data | Robust to incomplete datasets |
| Parallel & GPU Training (tree_method) | Optimizes computation | Faster training on large datasets |
| Early Stopping | Monitors validation error | Stops training when no further improvement |

**Table A3.** Techniques used in Transformer Decoder

| Technique | Purpose | Key Effect |
| --- | --- | --- |
| Feature Engineering (deltas, ratios, polynomials, rolling stats) | Enrich raw acoustic/musical descriptors | Captures nonlinear and temporal relationships |
| Normalization (meanstd scaling) | Standardizes feature ranges | Improves training stability and convergence |
| Feature Embeddings + Value Projection | Converts tabular features into tokenlike representations | Enables transformer to treat features as a sequence |
| Positional Encoding | Adds order information to feature tokens | Preserves structural context across features |
| Transformer Decoder Layers (multi‑head attention, feedforward, GELU) | Learns complex dependencies between features | Models nonlinear interactions in GPT‑style fashion |
| AdamW Optimizer + Cosine LR Scheduler | Modern training strategy | Enhances generalization and prevents overfitting |
| SmoothL1 Loss | Robust regression loss | Reduces sensitivity to outliers |
| Diagnostics (scatter, residuals, QQ, loss curves) | Visual evaluation of predictions | Validates model reliability and error distribution |

**Appendix B**

**Figure S1.** Cumulative Error Plot

Note: Figure S1 presents the cumulative error distribution for the Deep GRU model. Note: This diagnostic plot was generated on the original dataset and is provided for illustrative purposes to demonstrate error concentration behavior. Full cross-validation performance on the extended dataset is reported in Table 8.

**Figure S2.** Cumulative Error Plot (Tuned XGBoost)

Figure S2 presents the cumulative error distribution for the tuned XGBoost model. Note: This diagnostic plot was generated on the original dataset and is provided for illustrative purposes. Full cross-validation performance on the extended dataset is reported in Table 8.
